# Age-stratified adverse-event reporting signals of seven ALK/ROS1 tyrosine kinase inhibitors in FAERS: a disproportionality and time to onset study

**DOI:** 10.3389/fphar.2026.1891628

**Published:** 2026-09-07

**Authors:** Yongjian Liu, Yingyu Yu, Yige Yu, Xingjun Huang, Hao Zhong

**Affiliations:** 1 Department of Pharmacy, Huizhou Central People’s Hospital, Huizhou, China; 2 Department of Pharmacy, Shantou Central Hospital, Shantou, China; 3 Department of Pharmacy, The First Affiliated Hospital of Wenzhou Medical University, Wenzhou, China; 4 Department of Pharmacy, the Third Affiliated Hospital of Sun Yat-sen University, Guangzhou, China

**Keywords:** adverse-event reporting, age-stratified analysis, ALK/ROS1 tyrosine kinase inhibitors, disproportionality analysis, FAERS, pharmacovigilance

## Abstract

**Background:**

ALK/ROS1 tyrosine kinase inhibitors (TKIs) have improved outcomes in molecularly selected malignancies, but trial populations do not fully represent the age and clinical heterogeneity encountered in routine care. Comparative post-marketing evidence on age-related and drug-specific reporting patterns remains limited.

**Methods:**

We analyzed US Food and Drug Administration Adverse Event Reporting System data from 2004 Q1 through 2026 Q1. The study included 38,479 primary-suspect reports with known age for crizotinib, entrectinib, brigatinib, lorlatinib, repotrectinib, cabozantinib, or ceritinib. Reports were stratified as 0–17, 18–44, 45–64, or ≥65 years. A preferred term (PT) was considered positive only when prespecified criteria for ROR, PRR, BCPNN, and MGPS were all met. We evaluated formal drug-exposure-by-age interactions, drug-level PT-spectrum similarity, clinical priority, and reported time to onset (TTO) with Weibull modeling.

**Results:**

Positive PT counts were 64, 159, 230, and 178 across the four age groups, respectively, and 299 in the all-age analysis. Of 255 PTs eligible for formal interaction testing, 127 had an interaction false-discovery rate <0.05; 95 remained after exclusion of concepts not interpretable as adverse events. Descriptive displays highlighted pediatric skeletal concerns and neurologic or functional vulnerability in older reports, while formal contrasts distinguished these patterns from within-stratum signal detection. Drug-level positive-PT spectra showed generally low pairwise similarity, and target-set overlap correlated weakly with signal-spectrum similarity (Spearman ρ = 0.212, P = 0.356). TTO was evaluable for 15,810 reports (41.1%). Weibull estimates were below one across fitted pooled age groups, consistent with an early-failure reporting pattern.

**Conclusion:**

These findings support surveillance based on both age and the prescribed agent rather than a uniform class profile. The early reporting pattern favors close assessment soon after treatment initiation. FAERS signals remain hypothesis-generating and require confirmation in longitudinal patient-level data.

## 1 Introduction

Anaplastic lymphoma kinase (*ALK*) and *ROS1* rearrangements are clinically important oncogenic drivers, particularly in advanced non-small cell lung cancer (Soda et al., 2007; Shaw et al., 2014). Targeted inhibition has improved systemic disease control, expanded treatment options after resistance, and provided activity against central nervous system disease (Solomon et al., 2014; Shaw et al., 2020; Camidge et al., 2021; Drilon et al., 2024). As these agents are used across treatment lines and molecular contexts, clinical management increasingly depends on maintaining tolerability, preserving dose intensity, and recognizing adverse events promptly.

The evidence base for safety monitoring remains uneven across age groups. Registration trials of targeted anticancer drugs often enroll selected participants and may underrepresent frail older adults, patients with substantial comorbidities or polypharmacy, and children or adolescents treated in uncommon molecular settings (Ludmir et al., 2019). Real-world use encompasses broader variation in prior treatment, tumor burden, central nervous system involvement, organ reserve, and concomitant medication. Direct post-marketing comparisons among individual ALK/ROS1 TKIs remain sparse, and pooled all-age analyses may conceal variation in spontaneous reporting across age strata and agents (Wen et al., 2026; Yu et al., 2026).

We therefore examined FAERS reports for crizotinib, entrectinib, brigatinib, lorlatinib, repotrectinib, cabozantinib, and ceritinib. The study aimed to describe age-stratified reporting profiles, formally test age-related heterogeneity in reporting disproportionality, compare drug-specific positive-PT spectra, and characterize reported onset timing. The pooled seven-drug analysis was used as an aggregate screening layer, whereas single-drug, indication-context, sensitivity, and TTO analyses were used to support clinically cautious interpretation and monitoring (Leroy et al., 2014).

## 2 Materials and methods

### 2.1 Data source, report processing, and study design

**FIGURE 1:**
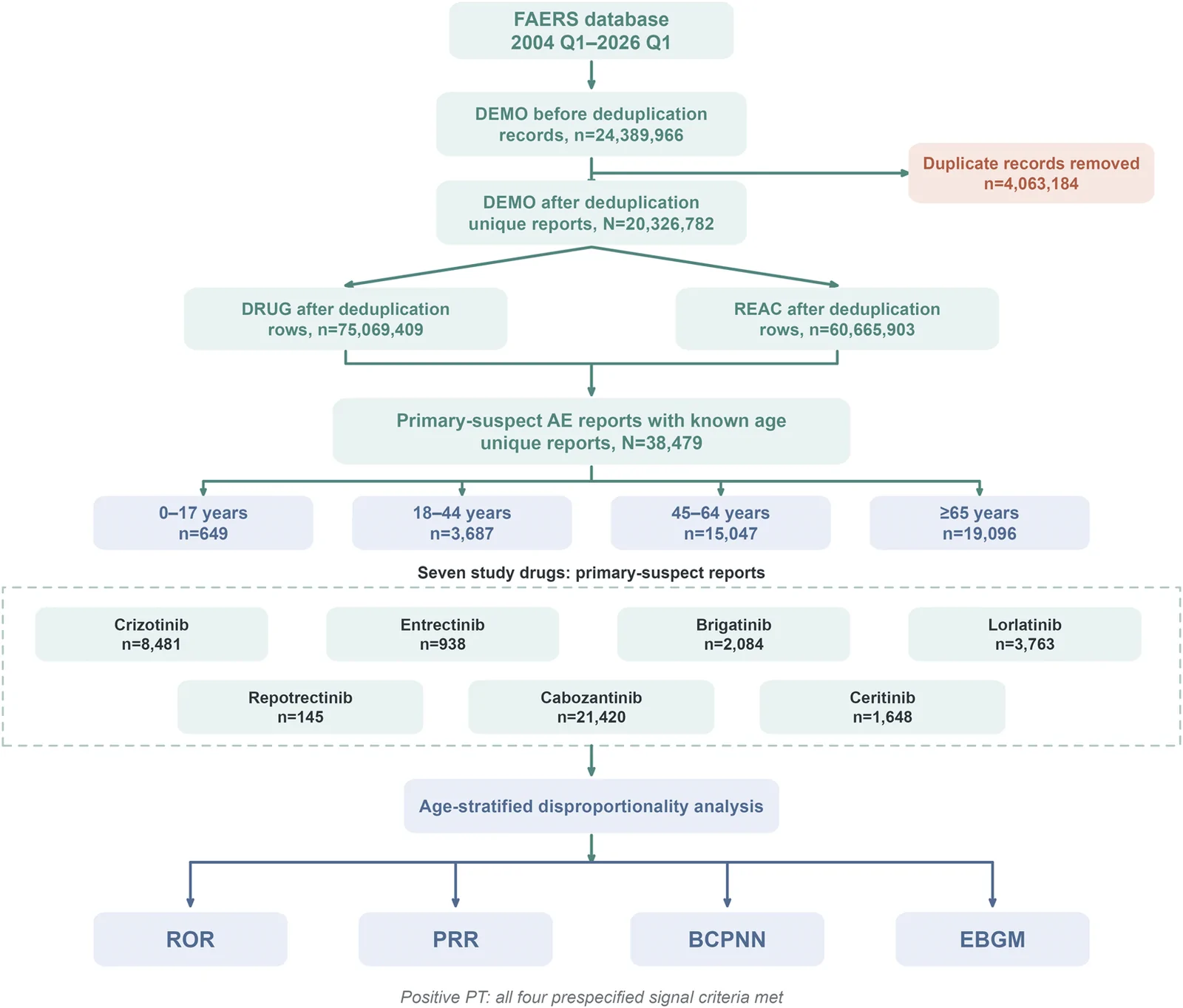
Study flow and signal-detection framework. FAERS reports submitted from 2004 Q1 through 2026 Q1 were deduplicated using CASEID, FDA_DT, and PRIMARYID. The pre‐deduplication DEMO count represents records, whereas the post-deduplication DEMO count represents unique reports; DRUG and REAC counts represent table rows. After restriction to primary-suspect (PS) reports for the seven study drugs and exclusion of records with unknown or missing age, 38,479 reports remained: 649 aged 0–17 years, 3,687 aged 18–44 years, 15,047 aged 45–64 years, and 19,096 aged ≥65 years. Signal detection used the reporting odds ratio (ROR), proportional reporting ratio (PRR), Bayesian confidence propagation neural network (BCPNN), and an operational empirical Bayes geometric mean (EBGM) measure. A preferred term (PT) was defined as positive only when all four prespecified signal criteria were met. Counts do not represent incidence, population risk, or causality.

This retrospective pharmacovigilance study used the US Food and Drug Administration Adverse Event Reporting System (FAERS) from 2004 Q1 through 2026 Q1. Demographic (DEMO), drug (DRUG), reaction (REAC), outcome (OUTC), indication (INDI), reporter-source (RPSR), and therapy (THER) files were linked using report identifiers. Duplicate reports were removed according to the FDA-recommended rule. Records were ordered by CASEID, FDA_DT, and PRIMARYID; for repeated CASEID values, the record with the latest FDA_DT was retained, and when CASEID and FDA_DT were identical, the record with the largest PRIMARYID was retained. This reduced the DEMO file from 24,389,966 to 20,326,782 reports, removing 4,063,184 duplicates. The deduplicated DRUG and REAC files contained 75,069,409 and 60,665,903 rows, respectively (Figure 1).

Reports listing crizotinib, entrectinib, brigatinib, lorlatinib, repotrectinib, cabozantinib, or ceritinib as the primary suspect drug were identified. Reports with unknown or missing age were excluded, and the remainder were classified as 0–17, 18–44, 45–64, or ≥65 years; an all-age cohort was retained for aggregate analyses. Reaction counts were based on unique PRIMARYID–PT pairs, so each report contributed no more than once to a given PT.

The pooled seven-drug cohort was used as an aggregate screening layer to characterize the age-stratified reporting landscape and identify PTs for further evaluation. It was not intended to estimate a uniform ALK/ROS1 TKI class effect. Because the agents differ in kinase selectivity, approved indications, treatment setting, market duration, and report volume, positive-PT profiles were also evaluated separately for each drug. Drug-specific analyses, pairwise signal-spectrum comparisons, and a sensitivity analysis excluding cabozantinib were used to assess dependence of the pooled findings on individual agents and indication mix.

### 2.2 MedDRA coding and baseline characteristics

Adverse events were coded at the Medical Dictionary for Regulatory Activities (MedDRA) PT and System Organ Class (SOC) levels using MedDRA version 28.1. Baseline characteristics were summarized by age group and for the all-age cohort, including sex, reporter type, seriousness, reported outcomes, and reporter country. Country abbreviations and equivalent full-name entries were harmonized before ranking by all-age report count. Reported outcome categories included death, hospitalization, life-threatening events, disability, congenital anomaly, and Other. For descriptive tabulation, each report was assigned to a single mutually exclusive outcome category.

### 2.3 Disproportionality measures and positive-signal definition

For every drug-age stratum and PT, a 2 × 2 table was constructed. Cell a denoted reports containing both the target drug and target event; b, reports containing the target drug and other events; c, reports containing other drugs and the target event; and d, reports containing other drugs and other events. Full formulas for the reporting odds ratio (ROR), proportional reporting ratio (PRR), Pearson chi-square statistic, Bayesian confidence propagation neural network information component (BCPNN/IC), multi-item gamma Poisson shrinker empirical Bayes geometric mean (MGPS/EBGM), confidence or credibility limits, continuity corrections, and positivity thresholds are provided in Supplementary Table S1.

ROR was positive when its lower 95% confidence bound exceeded 1; PRR was positive when PRR was ≥2 and chi-square was ≥4; BCPNN was positive when IC025 was >0; and MGPS was positive when EBGM05 was >2. Each criterion also required at least three target-drug reports. A PT was classified as positive only when all four algorithms met their prespecified criteria. This intersection definition was selected as a conservative screening rule to reduce algorithm-specific and sparse-count instability; it does not establish causality or incidence (Bate et al., 1998; Dumouchel, 1999; Evans et al., 2001; Van Puijenbroek et al., 2002; Sakaeda et al., 2014).

### 2.4 Descriptive SOC and PT profiling

SOC distributions were derived from age-stratified report counts. The main display was restricted to the 17 SOCs with the largest all-age counts. Because report volume varied substantially by age, stacked bars retained raw counts while a companion heatmap displayed the percentage contribution of each SOC within the corresponding stratum. Positive PT sets were compared descriptively using exclusive UpSet intersections. Age-stratified volcano plots displayed log_2_(ROR) against −log_10_(P value); eight clinically interpretable PTs per stratum were annotated using signal strength, report support, clinical relevance, FDA prescribing-information context, and age relevance. Drug-specific UpSet and lollipop displays were generated separately. PTs primarily reflecting disease progression, death, treatment failure, product problems, or other non-toxicity concepts were omitted from selected clinical displays but retained in complete tables.

### 2.5 Formal analysis of age-related reporting heterogeneity

Formal between-age heterogeneity was evaluated separately from descriptive signal detection within each age stratum. A PT was eligible for interaction modeling when it was positive in at least one age stratum, had at least 10 target-drug event reports across the four age groups, and occurred in at least two age groups. For each eligible PT, grouped binomial models used event and non-event counts from target-drug reports and all other FAERS drug reports. An additive model containing drug exposure and age group was compared by likelihood-ratio test with a model additionally containing the drug-exposure-by-age interaction. Interaction P values were adjusted across eligible PTs using the Benjamini–Hochberg false-discovery-rate procedure (Benjamini and Hochberg, 1995). Relative reporting odds ratios (rRORs) and 95% CIs were derived from the interaction model for the 0–17, 18–44, and ≥65-year groups relative to the 45–64-year reference group. These estimates quantify heterogeneity in reporting disproportionality and do not estimate age-specific incidence or causal effect modification.

### 2.6 Drug-level similarity and clinical priority

All-age positive-PT sets were extracted for each drug. Pairwise signal-spectrum similarity was measured using the Jaccard coefficient, defined as the number of shared positive PTs divided by the union of both positive-PT sets. Multidimensional scaling used one minus the Jaccard coefficient. Concise sets of key kinase targets were compiled for the seven agents, and target-set overlap was calculated using the same definition. Across the 21 drug pairs, the association between target-set and positive-PT-spectrum similarity was summarized by Spearman correlation. This analysis was exploratory because drug pairs share agents and are not statistically independent, and because the target lists were concise summaries rather than exhaustive kinome profiles.

Clinical priority was assigned only after four-algorithm positive-signal filtering. The exploratory framework, adapted from a previously published clinical-prioritization framework (Cecco et al., 2024), included reporting rate, consistency across disproportionality algorithms, the proportion of reports with death as an outcome, and DME/IME-based clinical relevance (Supplementary Table S2). It was used to order positive PTs for clinical review rather than estimate incidence, causality, or comparative risk. TTO was retained as an independent temporal dimension because onset dates were substantially incomplete and no externally validated weighting scheme combines TTO with the four score components. For each PT, temporal summaries included the number of valid TTO records, the median and interquartile range, the number and proportion with TTO ≤30 days, and a median-based temporal category (≤30, 31–90, or >90 days).

### 2.7 Time to onset and Weibull analyses

Time to onset (TTO) was calculated as the interval between reported treatment initiation and event onset. Reports without an evaluable date pair were counted separately; TTO ≤0 days and TTO >3,650 days were excluded. Empirical medians and interquartile ranges were the primary temporal summaries. Weibull shape parameters were estimated for pooled age groups, individual-drug all-age cohorts, drug-by-age strata, prespecified AE groups, and PT-specific clinical-priority rows when at least 30 valid TTO records were available. The minimum of 30 was a pragmatic, prespecified fitting threshold used to limit sparse-sample instability and should not be interpreted as a universal Weibull sample-size requirement. A β value < 1 was interpreted as an early-failure reporting pattern, β near 1 as a random-failure pattern, and β > 1 as a wear-out pattern. These reported-case distributions were not interpreted as population hazards or causal drug-by-age interactions. A conditional right-truncation analysis used the observable interval from treatment initiation to the database cutoff of 31 March 2026 (Leroy et al., 2014).

### 2.8 Additional sensitivity and exploratory subgroup analyses

Additional analyses were conducted to assess robustness and provide context for reporting heterogeneity while leaving the primary analysis unchanged. Strong CYP3A inhibitors and inducers were identified using the U.S. Food and Drug Administration resource Examples of Drugs that Interact with CYP Enzymes and Transporter Systems (U.S. Food and Drug Administration, 2026). Reports co-recording a non-index drug matching a strong CYP3A inhibitor or inducer were removed from both the target-drug cohort and the corresponding FAERS background. The four disproportionality algorithms were then recalculated for every pooled age stratum and individual-drug all-age cohort. Co-recording does not confirm overlapping administration; this was therefore treated as a conservative co-medication sensitivity analysis rather than a causal drug–drug interaction assessment.

Female and male signal profiles were evaluated within each age group and in the all-age cohort. Reporter-country analyses were conducted for the United States, Japan, China, and other known reporting countries; reports with country not specified were excluded from the four-group concordance comparison. These subgroup analyses were descriptive rather than confirmatory tests of effect modification. Cabozantinib indications were classified hierarchically as renal, hepatic, thyroid, primary lung, other malignant, non-malignant or unknown, or missing, and the pooled age-stratified analysis was repeated after cabozantinib was removed. Robustness was assessed using positive-PT retention, positive-set Jaccard similarity, and Spearman concordance of log_2_(ROR) estimates. The minimum report count was checked directly to verify that no positive PT was supported by a single target report.

### 2.9 Statistical analysis and software

Descriptive statistics were reported as counts and percentages. Unless otherwise specified, confidence intervals and statistical tests were two-sided. P values from PT-level age-interaction analyses were adjusted using the Benjamini–Hochberg procedure. Data preparation and large-scale case-level count extraction used Python 3.12.4 with DuckDB 1.4.1. Statistical analyses and figure generation used R 4.5.3, principally with data.table 1.18.2.1, dplyr 1.2.1, tidyr 1.3.2, readr 2.2.0, ggplot2 4.0.2, ggrepel 0.9.8, patchwork 1.3.2, and openxlsx 4.2.8.1. Because FAERS is a spontaneous-reporting system, all findings were interpreted as reporting signals rather than incidence estimates, absolute risks, comparative safety estimates, or evidence of causality.

## 3 Results

### 3.1 Study cohort and baseline characteristics

After deduplication and exclusion of reports with unknown or missing age, 38,479 individual case safety reports (ICSRs) identified one of the seven study drugs as the primary suspect. Report counts were 649 for patients aged 0–17 years, 3,687 for those aged 18–44 years, 15,047 for those aged 45–64 years, and 19,096 for those aged ≥65 years (Figure 1; Table 1). Cabozantinib contributed the largest number of reports, followed by crizotinib, lorlatinib, brigatinib, ceritinib, entrectinib, and repotrectinib. These counts reflect indication, market exposure, prescribing history, and report accrual rather than comparable exposure denominators (Supplementary Table S3).

**TABLE 1:** Baseline characteristics of primary-suspect adverse-event reports by age group.

Section	Characteristic	0–17 N = 649	18–44 N = 3687	45–64 N = 15047	≥65 N = 19096	All ages N = 38479
Overall	Reports, n	649	3,687	15,047	19,096	38,479
Sex	Female	280 (43.1%)	1739 (47.2%)	6149 (40.9%)	7006 (36.7%)	15174 (39.4%)
	Male	345 (53.2%)	1881 (51.0%)	8687 (57.7%)	11790 (61.7%)	22703 (59.0%)
	Missing	24 (3.7%)	67 (1.8%)	211 (1.4%)	300 (1.6%)	602 (1.6%)
Reporter type	Consumer	120 (18.5%)	1357 (36.8%)	6561 (43.6%)	7305 (38.3%)	15343 (39.9%)
	Health professional	105 (16.2%)	316 (8.6%)	1089 (7.2%)	1151 (6.0%)	2661 (6.9%)
	Physician	300 (46.2%)	1337 (36.3%)	4940 (32.8%)	7911 (41.4%)	14488 (37.7%)
	Pharmacist	45 (6.9%)	231 (6.3%)	1122 (7.5%)	1454 (7.6%)	2852 (7.4%)
	Missing/other	79 (12.2%)	446 (12.1%)	1335 (8.9%)	1275 (6.7%)	3135 (8.1%)
Outcome	Death	108 (16.6%)	1009 (27.4%)	3433 (22.8%)	3885 (20.3%)	8435 (21.9%)
	Hospitalization	197 (30.4%)	722 (19.6%)	2959 (19.7%)	3715 (19.5%)	7593 (19.7%)
	Life-threatening	22 (3.4%)	59 (1.6%)	246 (1.6%)	300 (1.6%)	627 (1.6%)
	Disability	6 (0.9%)	22 (0.6%)	82 (0.5%)	68 (0.4%)	178 (0.5%)
	Congenital anomaly	1 (0.2%)	3 (0.1%)	NA	NA	4 (0.0%)
	Other	315 (48.5%)	1872 (50.8%)	8327 (55.3%)	11128 (58.3%)	21642 (56.2%)
Seriousness	Yes	470 (72.4%)	2657 (72.1%)	9871 (65.6%)	11841 (62.0%)	24839 (64.6%)
	No	179 (27.6%)	1030 (27.9%)	5176 (34.4%)	7255 (38.0%)	13640 (35.4%)
Fatality	Yes	108 (16.6%)	1009 (27.4%)	3433 (22.8%)	3885 (20.3%)	8435 (21.9%)
	No	541 (83.4%)	2678 (72.6%)	11614 (77.2%)	15211 (79.7%)	30044 (78.1%)
Reporting country	United States	361 (55.6%)	1,397 (37.9%)	7,188 (47.8%)	8,891 (46.6%)	17,837 (46.4%)
	Japan	17 (2.6%)	307 (8.3%)	1,836 (12.2%)	5,188 (27.2%)	7,348 (19.1%)
	India	2 (0.3%)	448 (12.2%)	1,099 (7.3%)	495 (2.6%)	2,044 (5.3%)
	France	46 (7.1%)	184 (5.0%)	553 (3.7%)	675 (3.5%)	1,458 (3.8%)
	China	12 (1.8%)	145 (3.9%)	498 (3.3%)	305 (1.6%)	960 (2.5%)
	Canada	17 (2.6%)	100 (2.7%)	389 (2.6%)	354 (1.9%)	860 (2.2%)
	Italy	2 (0.3%)	89 (2.4%)	356 (2.4%)	383 (2.0%)	830 (2.2%)
	United Kingdom	46 (7.1%)	98 (2.7%)	264 (1.8%)	229 (1.2%)	637 (1.7%)
	Others	146 (22.5%)	919 (24.9%)	2864 (19.0%)	2576 (13.5%)	6505 (16.9%)

Values are n (%) unless otherwise specified. Percentages use the number of reports in each age stratum as the denominator. Outcome categories are mutually exclusive. The table presents the eight most frequently reported known countries ranked by all-age report count; the complete country distribution is supplied in the corresponding data sheet.

Male reports accounted for 59.0% of the all-age cohort, and their proportion increased across older strata. Consumers and physicians were the leading reporter categories. The United States and Japan contributed the largest numbers of reports, followed by India, France, and China; contributions from national reporting systems were highly unequal (Table 1).

Overall, 64.6% of reports were classified as serious. Death and hospitalization were the most frequently recorded specific serious outcomes, although their proportions varied among age strata. For descriptive tabulation, each report was assigned to a single outcome category. The observed distributions may reflect malignancy severity, treatment setting, indication, and reporting completeness rather than age-specific outcome risk (Table 1; Supplementary Figure S1).

### 3.2 Annual reporting trends

Annual reporting differed by drug, consistent with approval timing, indication mix, uptake, and accumulated market exposure. Cabozantinib had the largest cumulative reporting burden, whereas newer agents showed later increases and shorter observation periods. The 2026 data contain Q1 only and were displayed as a partial year rather than compared directly with complete calendar years (Supplementary Figure S2; Supplementary Table S3).

### 3.3 SOC distribution across age groups

**FIGURE 2:**
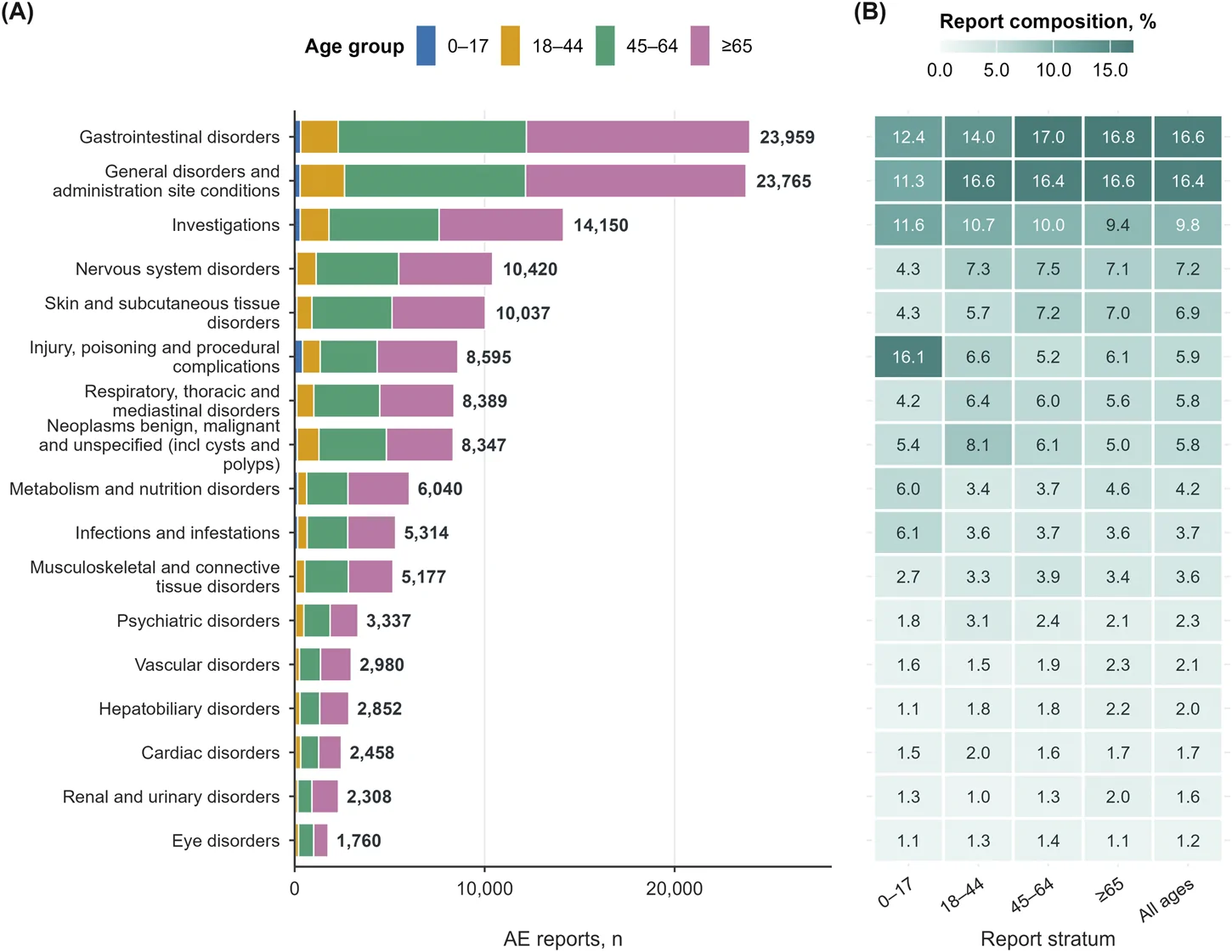
System organ class distribution across age groups. **(A)** Report counts for the 17 system organ classes (SOCs) with the largest all‐age counts, stratified by age group and ordered by the all-age total. Numbers at the ends of bars are all-age report counts. **(B)** Report composition within each displayed stratum; each cell gives the percentage of all SOC‐coded reports in that age cohort or in the all‐age comparator attributable to the corresponding SOC. Because only the leading 17 SOCs are displayed, percentages within a column are not expected to sum to 100%. SOC counts are report‐based and do not estimate incidence or comparative risk.

At the SOC level, gastrointestinal disorders, general disorders and administration-site conditions, investigations, nervous system disorders, and skin and subcutaneous tissue disorders accounted for the largest all-age report counts. Report-composition percentages showed that injury/procedural and investigation-related SOCs occupied a relatively larger share of pediatric reports, whereas gastrointestinal and general/systemic categories remained prominent in adult strata. These values describe the composition of submitted reports rather than age-specific incidence or comparative risk (Figure 2).

### 3.4 Positive PT signals and formal age-related reporting heterogeneity

Four-algorithm positive PTs numbered 64 in the 0–17-year group, 159 in the 18–44-year group, 230 in the 45–64-year group, and 178 in the ≥65-year group; 299 positive PTs were identified in the all-age pooled cohort (Supplementary Tables S4, S5). Consistent with the prespecified minimum-count criterion, every positive PT was supported by at least three target-drug reports. Twenty PTs were positive in all four age groups, whereas the remaining exclusive intersections showed more restricted descriptive patterns (Supplementary Figure S3; Supplementary Table S6). Volcano plots and single-drug displays provided complementary descriptive views of signal magnitude and report support, including clinically relevant skeletal, cardiopulmonary, metabolic, neurologic, mucocutaneous, and infectious domains (Supplementary Figure S4; Supplementary Figure S5A–G; Supplementary Table S7).

**FIGURE 3:**
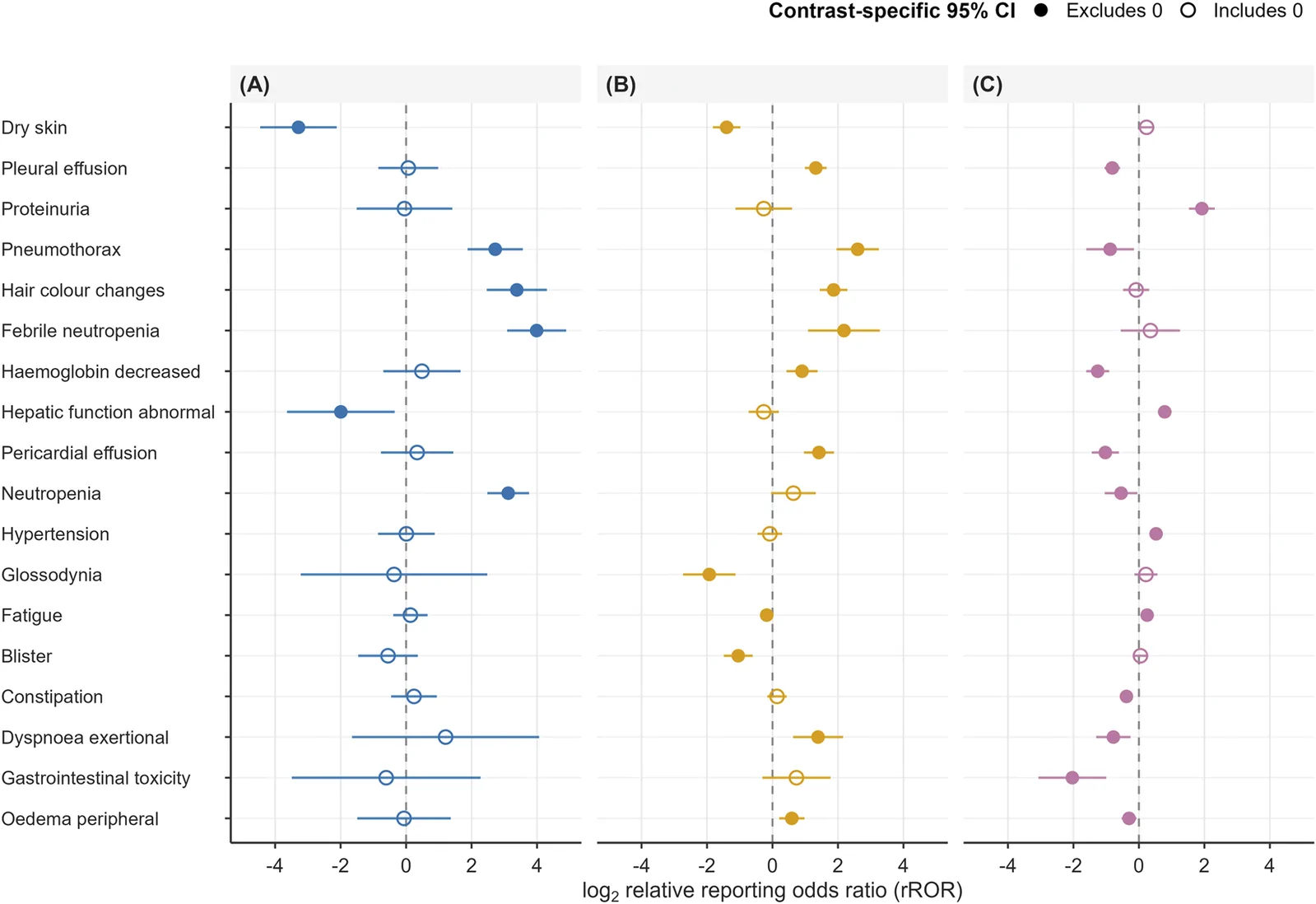
Age-related heterogeneity in PT reporting. The main display contains 18 clinically interpretable preferred terms (PTs) selected from those with a significant global drug-by-age interaction after false-discovery-rate correction (FDR <0.05). Selection was based on global interaction FDR and the largest absolute contrast estimate, while balancing the age contrast with the strongest departure; complete interaction results are provided in Supplementary Table S8. Relative reporting odds ratios (rRORs) are shown on the log_2_ scale for **(A)** 0–17 versus 45–64 years, **(B)** 18–44 versus 45–64 years, and **(C)** ≥65 versus 45–64 years; 45–64 years was the reference group. Horizontal lines indicate 95% confidence intervals (CIs), and the vertical dashed line denotes log_2_(rROR)=0. Solid points indicate that the 95% CI for that individual age contrast excluded 0; hollow points indicate that the PT passed the global interaction FDR criterion but the CI for that specific contrast included 0. Global interaction significance therefore should not be interpreted as evidence that every individual contrast is significant. These reporting contrasts do not establish incidence, risk, or causality.

Among 255 pooled-cohort PTs eligible for formal interaction modeling, 127 met the interaction FDR threshold of <0.05; 95 remained after excluding concepts that were not clinically interpretable as adverse events. Figure 3 displays 18 representative PTs selected by global interaction FDR and the largest absolute contrast estimate while balancing the age contrast with the strongest departure; complete results are provided in Supplementary Table S8 rROR estimates compared the 0–17, 18–44, and ≥65-year groups with the 45–64-year reference group. Accordingly, only formally supported differences were described as age-related reporting heterogeneity. Detection within one descriptive stratum without interaction evidence was not interpreted as an age-specific toxicity (Figure 3; Supplementary Table S8).

### 3.5 Drug-level signal spectra and clinical priority

**FIGURE 4:**
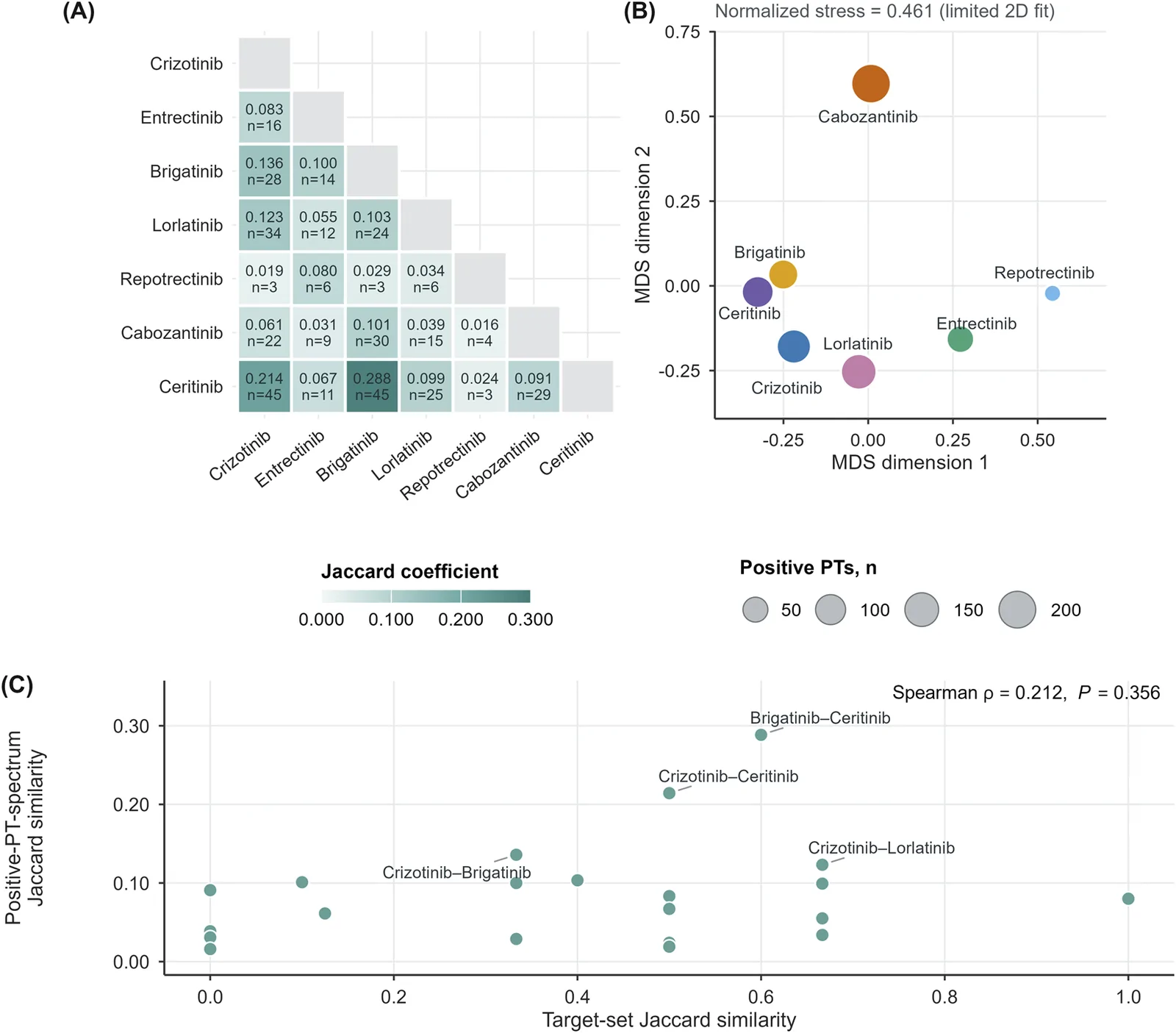
Similarity of positive‐PT spectra across the seven study drugs. **(A)** Lower‐triangular heatmap of pairwise Jaccard similarity between all‐age positive‐PT sets. Cells show the Jaccard coefficient and the number of shared PTs; diagonal cells are not evaluated. **(B)** Exploratory two-dimensional multidimensional scaling (MDS) representation of Jaccard dissimilarity. Point size indicates the number of all‐age positive PTs for each drug. MDS axes have no direct clinical unit; the normalized stress was 0.461, indicating limited two-dimensional fit. The panel is therefore provided only as an exploratory visual summary, and small differences in point-to-point distance should not be interpreted precisely. **(C)** Exploratory association between target‐set Jaccard similarity and positive‐PT‐spectrum Jaccard similarity across 21 drug pairs. No fitted regression model is shown because inference was based on Spearman rank correlation and the drug pairs are not statistically independent. Spearman ρ=0.212 and P=0.356. Similarity measures describe reporting spectra and do not imply equivalent toxicity, incidence, or causal relationships.

The size and composition of all-age positive-PT sets differed markedly among drugs. Pairwise Jaccard coefficients were generally low. The greatest similarities were observed for brigatinib–ceritinib (Jaccard = 0.288; 45 shared PTs) and crizotinib–ceritinib (Jaccard = 0.214; 45 shared PTs), whereas several pairs involving repotrectinib or cabozantinib had coefficients <0.05. The exploratory association between key-target overlap and positive-PT-spectrum similarity was weak and not statistically significant (Spearman ρ = 0.212, P = 0.356). Shared target sets therefore did not, by themselves, account for the observed reporting spectra (Figure 4; Supplementary Table S9).

PT-specific TTO information was available for 371 of 394 age-stratified medium/high-priority rows and 507 of 547 drug-stratified medium/high-priority rows. Among age-stratified medium/high-priority rows, 11/27 in the 0–17-year group, 15/74 in the 18–44-year group, 21/93 in the 45–64-year group, and 27/75 in the ≥65-year group had a median TTO ≤30 days. These annotations add temporal context to the clinical-review order without altering the four-component score or converting it into a validated prediction model (Supplementary Figure S6; Supplementary Tables S10,S11).

### 3.6 Time to onset and Weibull failure patterns

**FIGURE 5:**
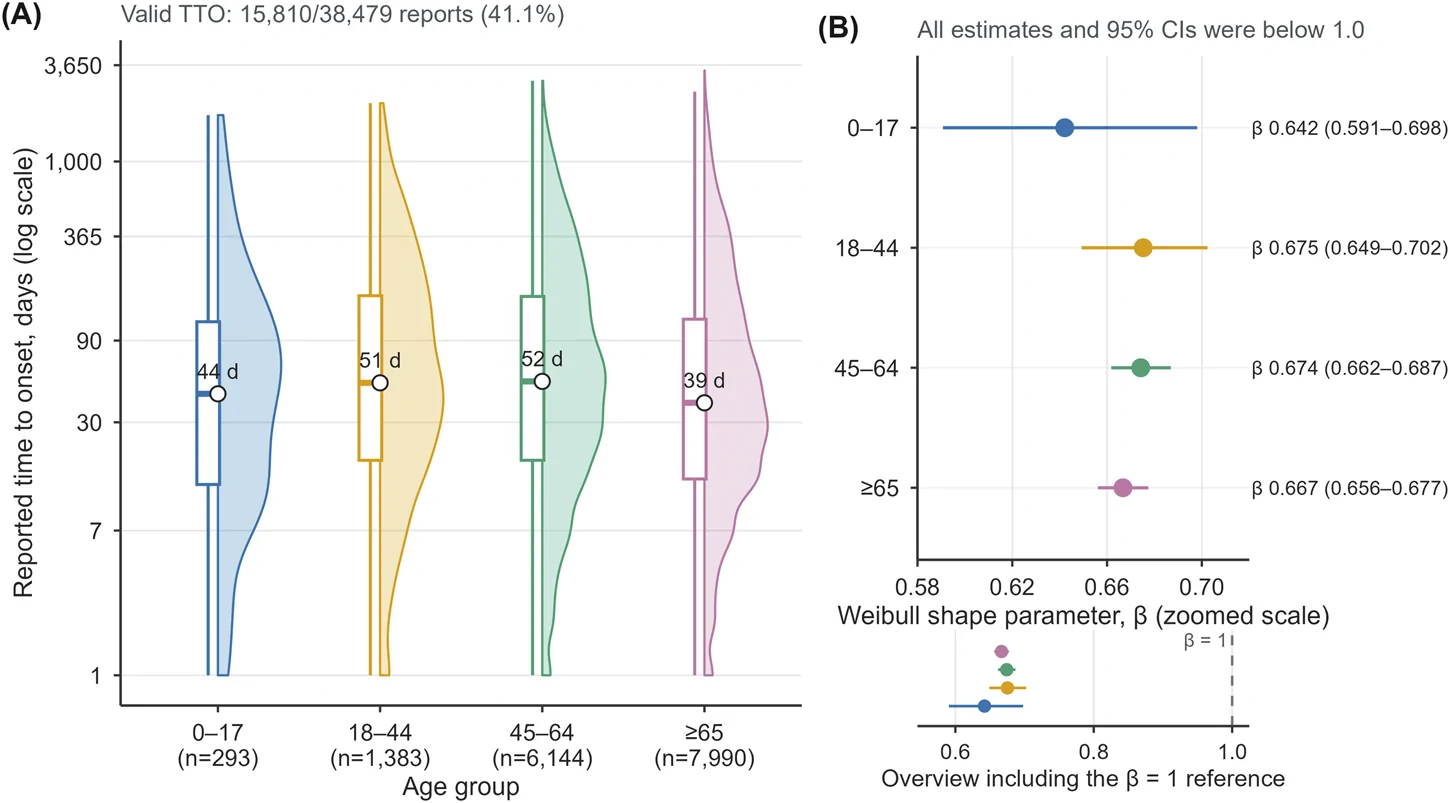
Reported time to onset and Weibull failure pattern by age. **(A)** Distribution of reports with valid time to onset (TTO) data, shown on a logarithmic day scale using half-violin plots with box plots and medians. TTO was calculable for 15,810 of 38,479 reports (41.1%); only records within the prespecified valid interval of 1–3,650 days are displayed. Sample sizes appear below each age group. **(B)** Weibull shape parameter (β) estimates and 95% CIs on a magnified scale, with a compact overview retaining the β=1 reference. All point estimates and CIs were below 1, consistent with an early-failure reporting pattern. Weibull models were fitted only when at least 30 valid TTO records were available. TTO reflects reported timing and should not be interpreted as a causal hazard function.

Of 38,479 primary-suspect reports, 22,667 lacked an evaluable treatment-start and event-onset date pair. Two additional reports had TTO >3,650 days; no report entering the TTO source dataset had TTO ≤0 days. The final analysis included 15,810 reports, or 41.1% of the primary cohort. Median reported TTO was 44 days overall and 44, 51, 52, and 39 days in the 0–17, 18–44, 45–64, and ≥65-year groups, respectively. The overall Weibull β was 0.668 (95% CI, 0.661–0.676), and all fitted pooled age-group estimates were <1, consistent with an early-failure reporting pattern. Conditional right-truncation analysis produced materially similar estimates (Figure 5; Supplementary Figure S7; Supplementary Table S12).

At the drug level, median TTO was 20 days for entrectinib, 42 days for cabozantinib, 48 days for crizotinib, 53 days for lorlatinib, 56 days for brigatinib, and 60 days for ceritinib. Repotrectinib had a median of 30.5 days but did not meet the prespecified minimum for Weibull fitting. Drug-by-age summaries showed additional descriptive variation. In the ≥65-year group, median TTO ranged from 14 days for entrectinib to 49 days for brigatinib; corresponding medians were 34 days for ceritinib, 37 days for crizotinib, 40 days for cabozantinib, and 43.5 days for lorlatinib. Repotrectinib had a median of 7.5 days in this stratum, but only 10 valid records were available. Across prespecified clinically relevant AE groups, fitted β estimates were also <1. These values describe timing among submitted cases and not comparative incidence or true latency distributions (Supplementary Figures S8, S9; Supplementary Table S12).

### 3.7 Sensitivity and exploratory subgroup analyses

Exclusion of reports co-recording strong CYP3A inhibitors or inducers removed 153 of 38,479 target-drug reports (0.40%). Across pooled age strata, positive-set Jaccard coefficients ranged from 0.899 to 0.994 and Spearman correlations for log_2_(ROR) ranged from 0.998 to 0.999. Individual-drug all-age Jaccard coefficients ranged from 0.957 to 1.000, indicating little change in the main signal profiles under this restriction (Supplementary Figure S10; Supplementary Table S13).

The sex-stratified all-age analysis included 15,174 female and 22,703 male reports. The female–male positive-set Jaccard coefficient was 0.457 overall and ranged from 0.294 to 0.408 across age strata. Reporter-country analyses included 17,837 reports from the United States, 7,348 from Japan, 960 from China, and 12,045 from other known countries; 289 reports with country not specified were excluded from the four-group comparison. Pairwise country Jaccard coefficients ranged from 0.064 to 0.231. These descriptive differences may reflect sample size, indication mix, drug access, market history, and reporting practice and were not interpreted as biological effect modification (Supplementary Figure S11; Supplementary Table S13).

Cabozantinib accounted for 21,420 of 38,479 reports (55.7%). Renal cancer accounted for 65.0% of its reports, hepatocellular or other hepatic cancer for 10.5%, thyroid cancer for 5.3%, and primary lung cancer or NSCLC for 1.1%. Removing cabozantinib reduced the all-age positive-set Jaccard coefficient to 0.392 and retained 51.8% of primary pooled positive PTs. The effect was greatest in the ≥65-year group (Jaccard = 0.239), demonstrating material dependence of the pooled profile on cabozantinib and its indication mix (Supplementary Figures S10, S11; Supplementary Table S13).

## 4 Discussion

The revised analysis separates detection of a signal within an age stratum from evidence that reporting disproportionality differs between age groups. The former is useful for signal discovery, whereas the latter requires a formal interaction analysis. Of the 255 PTs eligible for modeling, 127 met the interaction FDR threshold, and 95 remained clinically interpretable after exclusion of non-toxicity concepts. The manuscript therefore uses “age-stratified reporting signal” for within-stratum findings and reserves “age-related reporting heterogeneity” for globally supported interaction results; a significant global interaction does not imply that every individual age contrast is significant (Figure 3; Supplementary Table S8). Neither term implies incidence, comparative risk, or causal susceptibility, consistent with current guidance for reporting disproportionality analyses (Fusaroli et al., 2024).

The pooled cohort provides an efficient initial screen but should not be read as a uniform ALK/ROS1 TKI class profile. Cabozantinib contributed more than half of all reports and was predominantly reported for renal, hepatic, and thyroid malignancies; primary lung cancer or NSCLC represented only 1.1%. Its removal substantially altered the pooled positive-PT set, particularly among older reports (Supplementary Figures S10 and S11; Supplementary Table S13). These findings are compatible with confounding by indication and channeling, because disease burden, treatment line, previous therapy, and supportive care can influence spontaneous reporting independently of pharmacology (Petri and Urquhart, 1991; Sendor and Stürmer, 2022). Clinical interpretation should therefore place greater weight on the single-drug results, with the pooled analysis retained as an aggregate screening layer.

Drug-level similarity also argues against a single toxicity profile. Several ALK-directed pairs shared more positive PTs than most other pairs, but concise target overlap explained little of the variation in signal-spectrum similarity (Figure 4; Supplementary Table S9). Kinase selectivity remains relevant, yet indication, dose, treatment duration, central nervous system penetration, off-target activity, market maturity, and report volume also shape the observed spectra. Cabozantinib inhibition of VEGFR, MET, and AXL provides a pharmacologic basis for vascular, mucosal, skin, and wound-healing concerns, whereas lorlatinib’s central nervous system penetration is relevant to neurologic monitoring and its established metabolic toxicity (Yakes et al., 2011; Shaw et al., 2020; Zhou et al., 2023). The weak target–signal correlation indicates that concise target lists alone are insufficient; it does not imply that pharmacology is unimportant.

Some descriptive age-stratified findings remain clinically useful when considered with drug pharmacology and patient context. Pediatric musculoskeletal and fracture-related signals warrant scrutiny in reports involving TRK-inhibiting agents because neurotrophin/TRK signaling contributes to sensory innervation, osteoblast and osteoclast regulation, mineralization, and growth-plate homeostasis (Su et al., 2018; Kim et al., 2023). These reports are nevertheless vulnerable to confounding by malignancy, corticosteroid exposure, immobility, malnutrition, radiotherapy, and pre-existing skeletal fragility. In older adults, reduced hepatic and renal reserve, altered body composition, polypharmacy and CYP3A competition, baseline cognitive vulnerability, and immunosenescence may lower the threshold for neurologic, infectious, skin, or impaired-healing events (Mangoni and Jackson, 2004; Van Leeuwen et al., 2014). These mechanisms support closer review but do not convert reporting associations into proven age-specific toxicities.

Clinical priority and TTO address complementary questions. The priority score orders positive PTs by reporting burden, algorithmic consistency, death-outcome proportion, and medical importance; TTO describes when events tended to be reported among cases with usable dates (Supplementary Figure S6; Supplementary Tables S2, S10, S11). Adding an unvalidated TTO weight would make the score dependent on substantial date missingness and an arbitrary weighting decision. Reporting PT-specific timing alongside the original score is more transparent. Medium/high-priority PTs with early median onset may reasonably receive earlier monitoring attention, but neither priority nor timing establishes incidence or causality (Leroy et al., 2014; Cecco et al., 2024).

The consistent Weibull estimates below one support closer surveillance early after treatment initiation (Figure 5; Supplementary Figures S7–S9; Supplementary Table S12). Baseline review should include concomitant medications, organ function, and agent-specific toxicity risks. Symptoms and relevant laboratory measurements may warrant frequent reassessment during the first month, followed by monitoring tailored to the prescribed drug and toxicity domain. Drug-by-age summaries suggest that reported timing in older adults was not identical across agents, but these differences remain descriptive because FAERS contains reported cases rather than complete exposed risk sets. The right-truncation analysis reduced concern that the overall early-failure pattern was solely an artifact of unequal observation time, without converting the estimates into causal hazard functions (Leroy et al., 2014).

Excluding reports co-recording strong CYP3A modulators produced little change in the principal signal spectra (Supplementary Figure S10; Supplementary Table S13). This supports numerical robustness but does not rule out clinically important interactions, because co-recording does not establish simultaneous exposure, dose, adherence, or treatment duration (Shao et al., 2014; Van Leeuwen et al., 2014). Sex and reporter-country analyses showed greater descriptive heterogeneity (Supplementary Figure S11; Supplementary Table S13), but biological differences cannot be separated from sample size, indication, drug availability, market history, and reporting behavior. These findings provide context for reporting heterogeneity rather than comparative risk estimates.

Several limitations remain. FAERS lacks exposure denominators and is affected by under-reporting, stimulated and notoriety reporting, incomplete dates and covariates, duplicate clinical narratives, and the Weber effect for newer agents (Hartnell and Wilson, 2004; Pariente et al., 2007; Fusaroli et al., 2024). Disproportionality cannot estimate incidence or prove causality. Indication- and treatment-line channeling may inflate progression, death, neurologic, or complication terms among patients with advanced disease. The subgroup analyses were exploratory, and strata had unequal report support. TTO estimates applied to only 41.1% of the primary cohort and may remain affected by date quality despite the right-truncation analysis. The concise target sets were not exhaustive kinome profiles, and pairwise drug comparisons were not statistically independent. These findings should therefore be treated as hypothesis-generating and validated in longitudinal datasets containing patient-level exposure, dose, comorbidity, concomitant treatment, and follow-up.

## 5 Conclusion

The post-marketing safety of these seven kinase inhibitors cannot be adequately represented by a single pooled class description. Formal age comparisons, drug-specific reporting spectra, indication context, and reported onset timing provide distinct information for pharmacovigilance. The consistent early-failure pattern supports closer surveillance soon after treatment initiation, followed by monitoring adapted to the prescribed agent and the patient’s clinical context. These spontaneous-reporting findings generate testable safety hypotheses rather than comparative risk estimates and require confirmation in longitudinal patient-level datasets.

## Data Availability

The FAERS data analyzed in this study are publicly available from the U.S. Food and Drug Administration. Derived analysis tables and figure source data are provided in the Supplementary Material. Further inquiries may be directed to the corresponding author.
