# Supplementary material for: Age-stratified adverse-event reporting signals of seven ALK/ROS1 tyrosine kinase inhibitors in FAERS: a disproportionality and time to onset study
Source: Front Pharmacol. 2026 Sep 7;17:1891628. doi: 10.3389/fphar.2026.1891628 (PMC13590430; doi:10.3389/fphar.2026.1891628)
Supplement: Supplementary file 1 [file Supplementaryfile1.docx]

**Supplementary figures**

**Figure S1. Serious outcome burden by age group.** Percentages of PS reports recording death, hospitalization, a life-threatening outcome, or disability are shown for the four age strata and all ages. The denominator for each percentage is the number of PS reports in that age cohort. Serious outcomes are not mutually exclusive; therefore, category percentages should not be summed. Reported outcomes do not estimate outcome incidence or drug-attributable risk.

**Figure S2. Annual PS adverse-event report counts by drug.** Annual counts are shown from 2011 through 2025, with 2026 Q1 displayed separately on a shaded background. The 2025-to-2026 Q1 segments are dashed because 2026 contains only the first quarter and is not directly comparable with complete calendar years; no annualization was performed. The y-axis uses a square-root scale. Labels are limited to each drug’s peak, the latest complete year, and 2026 Q1. Temporal changes may reflect uptake, indication expansion, publicity, regulatory activity, and reporting practices rather than changes in incidence.

**Figure S3. Exclusive intersections of positive PTs across age groups in the pooled seven-drug analysis.** Bars show the number of positive PTs present in exactly the combination of age groups indicated by the connected colored dots. Thus, each PT contributes to one mutually exclusive intersection rather than to every nested subset. The complete identities and PT-level statistics are provided in the supplementary tables. Intersection sizes describe overlap in reported signal sets, not comparative incidence or risk.

**Figure S4. Age-stratified volcano plots of disproportionality signals.** Each panel plots log₂(ROR) against −log₁₀(P value) for (A) 0–17 years, (B) 18–44 years, (C) 45–64 years, (D) ≥65 years, and (E) all ages. Colored points denote positive PTs, and the eight prespecified annotations in each panel were selected using statistical strength together with age-specific clinical relevance. Values above the panel-specific 99.5th percentile of −log₁₀(P) were visually capped and marked by upward triangles; the underlying P values were not altered. Labels identify PTs for interpretation rather than a separate significance threshold. Complete PT-level results are retained in the supplementary tables. Disproportionality signals indicate reporting associations, not incidence, population risk, or causality.

**Figure S5A. Age-group overlap and selected age-specific positive PTs for crizotinib.** The upper display shows exclusive UpSet intersections across the four age groups; each bar counts PTs present in exactly the age-group combination indicated by the connected colored dots. The lower display shows up to four highest-ROR PTs from each prespecified display category, with a maximum of 20 PTs. The x-axis is log₂(ROR); labels report the untransformed ROR and target-event report count (n). The full signal set is retained in the supplementary tables. High ROR estimates based on small n may be unstable and warrant cautious interpretation.

**Figure S5B. Age-group overlap and selected age-specific positive PTs for entrectinib.** Upper and lower display definitions and interpretation are as described for Figure S5A. Small or absent intersections reflect the limited number of positive PTs and should be interpreted descriptively.

**Figure S5C. Age-group overlap and selected age-specific positive PTs for brigatinib.** Upper and lower display definitions and interpretation are as described for Figure S5A.

**Figure S5D. Age-group overlap and selected age-specific positive PTs for lorlatinib.** Upper and lower display definitions and interpretation are as described for Figure S5A.

**Figure S5E. Age-group overlap and selected age-specific positive PTs for repotrectinib.** Upper and lower display definitions and interpretation are as described for Figure S5A. Because several estimates are based on very small target-event counts, the displayed RORs are exploratory.

**Figure S5F. Age-group overlap and selected age-specific positive PTs for cabozantinib.** Upper and lower display definitions and interpretation are as described for Figure S5A.

**Figure S5G. Age-group overlap and selected age-specific positive PTs for ceritinib.** Upper and lower display definitions and interpretation are as described for Figure S5A.

**Figure S6. Clinical-priority composition of positive PTs by drug and age group.** Cells show the number of medium/high-priority positive PTs divided by all positive PTs and the corresponding percentage. Fill represents this percentage. Cells based on fewer than 30 positive PTs are visually muted to limit the influence of small denominators; an asterisk and dashed border identify denominators of 10 or fewer, which should be interpreted cautiously. Grey cells are not estimable and are distinct from a true zero. Priority categories were assigned using the prespecified clinical-prioritization score. The display supports triage of signals and is not a measure of incidence or causal severity.

**Figure S7. Sensitivity analysis for right truncation in Weibull estimation.** Ordinary Weibull β estimates are compared with right-truncation-corrected estimates for pooled age groups and individual drugs in the all-age cohort. Horizontal lines indicate 95% CIs, and the dashed line denotes β=1. Repotrectinib was not fitted because only 26 valid TTO records were available; the prespecified minimum was n=30. Concordant estimates below 1 support an early-failure reporting pattern, but the analysis remains descriptive and does not estimate causal hazards.

**Figure S8. TTO and Weibull estimates by drug and age group.** (A) Median reported TTO in days and the number of valid TTO records for each drug-age cell. Cells with fewer than 30 records are visually muted. (B) Weibull β estimates and valid TTO sample sizes. Models were not fitted when n<30, and these cells are explicitly marked “Not fitted”; missing estimates are not encoded as zero. All-age columns are shown for comparison. Small-sample cells should be interpreted descriptively.

**Figure S9. TTO characteristics of clinically relevant adverse-event groups in the all-age cohort.** (A) Median reported TTO and interquartile range for each prespecified adverse-event group. (B) Weibull β estimates with 95% CIs on a magnified scale and an overview retaining the β=1 reference. All estimates and 95% CIs were below 1.0. Only reports with calculable TTO within 1–3,650 days were included, and Weibull fitting required at least 30 valid records. These patterns describe reporting time and do not establish causal hazards.

**Figure S10. Signal-set sensitivity analyses.** (A) Concordance with the primary analysis after excluding reports involving strong CYP3A modulators, summarized by positive-set Jaccard similarity and Spearman correlation of log₂(ROR) values across pooled age cohorts and individual drugs. The x-axis is magnified from 0.85 to 1.00, and the dashed line denotes perfect concordance. (B) Concordance of the pooled seven-drug analysis after excluding cabozantinib, summarized by positive-set Jaccard similarity and retention of primary signals. Values nearer 1 indicate greater concordance. These secondary analyses assess robustness of the reporting pattern and do not remove residual confounding.

**Figure S11. Exploratory stratified and indication-context analyses.** (A) Female-male concordance within each age cohort, summarized by positive-set Jaccard similarity (blue) and Spearman correlation of log₂(ROR) values (grey). (B) Pairwise Jaccard similarity of positive-PT sets across reporter-country strata; diagonal values equal 1 by definition, and missing values are visually distinct from true zeros. US denotes the United States, and Other denotes other known reporter countries. (C) Distribution of recorded indication contexts among cabozantinib reports. These analyses describe the available reports and remain susceptible to missing data, reporting heterogeneity, channeling, and confounding by indication.
