# Supplementary material for: Age-stratified adverse-event reporting signals of seven ALK/ROS1 tyrosine kinase inhibitors in FAERS: a disproportionality and time to onset study
Source: Front Pharmacol. 2026 Sep 7;17:1891628. doi: 10.3389/fphar.2026.1891628 (PMC13590430; doi:10.3389/fphar.2026.1891628)
Supplement: Supplementary file 2 [file Table1.docx]

**Supplementary tables**

**Table S1.** Criteria and formulas for the four disproportionality algorithms.

**Table S1a.** Definitions of the two-by-two contingency table for disproportionality analysis.

| **Item** | **Target adverse events reported** | **Other adverse events reported** | **Total** |
| --- | --- | --- | --- |
| Target drug(s) | a | b | a + b |
| Other drugs | c | d | c + d |
| Total | a + c | b + d | a + b + c + d |

**Note**: N denotes the total number of reports in the analysis stratum.

**Table S1b.** Calculation formulas and positive-signal criteria for ROR, PRR, BCPNN, and operational EBGM.

| **Algorithm** | **Estimate** | **Confidence/shrinkage limit** | **Positive-signal criterion** | **Minimum report criterion** |
| --- | --- | --- | --- | --- |
| ROR | ROR = (a × d)/(b × c) SE[ln(ROR)] = √(1/a + 1/b + 1/c + 1/d) | 95% CI = exp{ln(ROR) ± 1.96 × SE[ln(ROR)]} | Lower bound of the 95% CI > 1 | Target-event reports ≥3 |
| PRR | PRR = [a/(a+b)]/[c/(c+d)] Pearson χ² = N(ad−bc)²/[(a+b)(c+d)(a+c)(b+d)] No Yates continuity correction was applied. | Not applicable | PRR ≥ 2 and Pearson χ² ≥ 4 | Target-event reports ≥3 |
| BCPNN | IC = log₂{aN/[(a+b)(a+c)]} Prior settings: α₁ = β₁ = γ₁₁ = 1; α = β = 2 γ = γ₁₁(N+α)(N+β)/[(a+b+α₁)(a+c+β₁)] E(IC) = log₂{(a+γ₁₁)(N+α)(N+β)/[(N+γ)(a+b+α₁)(a+c+β₁)]} V(IC) = 1/(ln 2)² × [(N−a+γ−γ₁₁)/{(a+γ₁₁)(1+N+γ)} + (N−a−b+α−α₁)/{(a+b+α₁)(1+N+α)} + (N−a−c+β−β₁)/{(a+c+β₁)(1+N+β)}] IC025 = E(IC) − 2 × √V(IC) | IC025 = E(IC) − 2 × √V(IC) | IC025 > 0 | Target-event reports ≥3 |
| Operational EBGM | Expected count E = (a+b)(a+c)/N EBGM = a/E = aN/[(a+b)(a+c)] SE[ln(EBGM)] = √(1/a + 1/b + 1/c + 1/d) | EBGM05 = exp{ln(EBGM) − 1.96 × SE[ln(EBGM)]} | EBGM05 > 2 | Target-event reports ≥3 |

**Note:** Pearson χ² was used for PRR signal detection; no continuity correction was applied. A PT was positive only when all four criteria were met.

**Table S2.** Criteria for the exploratory clinical-priority framework.

| **Criterion** | **2 points** | **1 point** | **0 points** | **Role in this study** |
| --- | --- | --- | --- | --- |
| Reporting rate (cases/non-cases) | >10% | 1-10% | <1% | Ranks the concentration of the PT among other reported PTs for the same cohort. |
| Signal stability (consistency across disproportionality analyses) | All four algorithms positive | Not applicable after the four-algorithm eligibility filter | Not applicable after the four-algorithm eligibility filter | Confirms eligibility. Because all retained PTs were positive in all four algorithms, this component was constant and did not discriminate within the positive set. |
| Reported case fatality rate (deaths among PT reports) | >50% | 25-50% | <25% | Adds reported outcome severity; it does not estimate a causal case-fatality risk. |
| Clinical relevance | DME | IME | Neither DME nor IME | Adds regulatory clinical importance based on DME/IME classification. |

**Note:** Total scores were categorized as high (≥6), medium (4–5), or low (≤3). The framework prioritizes signals for clinical review and is not a validated measure of incidence, risk, or causality.

**Table S3.** Drug observation maturity and report support for the seven study drugs.

| **Drug** | **Initial US approval date** | **First observed quarter** | **Observable quarters through 2026 Q1** | **PS reports with known age, n** | **Positive PTs, n** | **Valid TTO records, n** | **TTO retention, %** | **Key targets** | **FDA-approved indication(s)** |
| --- | --- | --- | --- | --- | --- | --- | --- | --- | --- |
| Crizotinib | 2011-08-26 | 2011 Q3 | 59 | 8,481 | 144 | 2,773 | 32.7 | ALK; ROS1; MET | ALK- or ROS1-positive NSCLC; ALK-positive pediatric/young-adult ALCL and IMT |
| Cabozantinib | 2012-11-29 | 2012 Q1 | 57 | 21,420 | 237 | 9,555 | 44.6 | VEGFR2; MET; AXL; RET; KIT; FLT3 | Multiple solid tumors, including RCC, HCC, differentiated or medullary thyroid cancer |
| Ceritinib | 2014-04-29 | 2012 Q3 | 55 | 1,648 | 111 | 977 | 59.3 | ALK; IGF-1R; ROS1 | ALK-positive metastatic NSCLC |
| Brigatinib | 2017-04-28 | 2017 Q2 | 36 | 2,084 | 90 | 997 | 47.8 | ALK; ROS1; IGF-1R; FLT3; mutant EGFR | ALK-positive metastatic NSCLC |
| Lorlatinib | 2018-11-02 | 2017 Q1 | 37 | 3,763 | 166 | 1,081 | 28.7 | ALK; ROS1 | ALK-positive metastatic NSCLC |
| Entrectinib | 2019-08-15 | 2019 Q2 | 28 | 938 | 64 | 401 | 42.8 | TRKA/B/C; ROS1; ALK | ROS1-positive NSCLC and NTRK fusion-positive solid tumors |
| Repotrectinib | 2023-11-15 | 2024 Q1 | 9 | 145 | 17 | 26 | 17.9 | ROS1; TRKA/B/C; ALK | ROS1-positive NSCLC and NTRK fusion-positive solid tumors |

**Note:** Approval dates and indication contexts were abstracted from FDA prescribing information; first observed quarter was derived from FAERS source-file identifiers.Abbreviations: FAERS, FDA Adverse Event Reporting System; FDA, U.S. Food and Drug Administration; PS, primary suspect; PT, preferred term; TTO, time to onset.

**Table S4.** Age-stratified positive preferred-term signals in the pooled seven-drug cohort.

The complete table is provided in Supplementary Data Sheet 1, worksheet “Table S4”.

**Table S5.** Complete all-age positive preferred-term signal table for the pooled cohort and individual drugs.

The complete table is provided in Supplementary Data Sheet 1, worksheet “Table S5”.

**Table S6.** Positive preferred-term signals shared across all four age groups.

| **PT** | **SOC** | **0–17 n** | **0–17 ROR (95%CI)** | **18–44 n** | **18–44 ROR (95%CI)** | **45–64 n** | **45–64 ROR (95%CI)** | **≥65 n** | **≥65 ROR (95%CI)** | **All ages n** | **All ages ROR (95%CI)** |
| --- | --- | --- | --- | --- | --- | --- | --- | --- | --- | --- | --- |
| Diarrhoea | Gastrointestinal disorders | 56 | 3.69 (2.83-4.81) | 382 | 3.66 (3.31-4.05) | 2,194 | 3.57 (3.42-3.73) | 3,060 | 3.53 (3.41-3.66) | 5,692 | 3.87 (3.77-3.97) |
| Decreased appetite | Metabolism and nutrition disorders | 37 | 4.26 (3.08-5.90) | 153 | 3.92 (3.34-4.59) | 963 | 4.33 (4.06-4.62) | 1,696 | 4.39 (4.18-4.61) | 2,849 | 4.76 (4.58-4.94) |
| Malignant neoplasm progression | Neoplasms benign, malignant and unspecified (incl cysts and polyps) | 48 | 36.62 (27.37-49.00) | 274 | 29.57 (26.17-33.42) | 882 | 9.04 (8.45-9.67) | 741 | 4.77 (4.43-5.13) | 1,945 | 8.61 (8.23-9.01) |
| Palmar-plantar erythrodysaesthesia syndrome | Skin and subcutaneous tissue disorders | 12 | 53.47 (29.83-95.83) | 102 | 50.74 (41.45-62.11) | 725 | 26.73 (24.75-28.88) | 1,070 | 34.38 (32.21-36.71) | 1,909 | 36.50 (34.79-38.30) |
| Constipation | Gastrointestinal disorders | 17 | 4.01 (2.49-6.47) | 109 | 3.74 (3.09-4.51) | 631 | 3.39 (3.14-3.67) | 809 | 2.61 (2.43-2.80) | 1,566 | 3.31 (3.15-3.48) |
| Neoplasm progression | Neoplasms benign, malignant and unspecified (incl cysts and polyps) | 28 | 34.56 (23.65-50.50) | 229 | 57.87 (50.50-66.31) | 588 | 14.39 (13.24-15.64) | 602 | 9.90 (9.12-10.75) | 1,447 | 15.64 (14.83-16.49) |
| Stomatitis | Gastrointestinal disorders | 17 | 8.05 (4.99-13.00) | 88 | 7.49 (6.07-9.25) | 555 | 9.45 (8.68-10.29) | 757 | 10.01 (9.30-10.77) | 1,417 | 9.97 (9.45-10.51) |
| Disease progression | General disorders and administration site conditions | 21 | 6.33 (4.11-9.74) | 177 | 11.72 (10.10-13.62) | 568 | 4.89 (4.50-5.31) | 614 | 3.99 (3.68-4.32) | 1,380 | 5.26 (4.99-5.55) |
| Blister | Skin and subcutaneous tissue disorders | 10 | 4.63 (2.48-8.62) | 47 | 3.28 (2.46-4.38) | 372 | 6.78 (6.12-7.52) | 407 | 7.01 (6.35-7.74) | 836 | 6.31 (5.89-6.76) |
| Pleural effusion | Respiratory, thoracic and mediastinal disorders | 10 | 4.79 (2.57-8.92) | 103 | 11.42 (9.39-13.89) | 274 | 4.57 (4.05-5.15) | 294 | 2.60 (2.32-2.92) | 681 | 4.17 (3.86-4.49) |
| Hypothyroidism | Endocrine disorders | 15 | 17.96 (10.76-29.98) | 51 | 9.27 (7.03-12.23) | 208 | 6.31 (5.50-7.24) | 365 | 8.92 (8.03-9.91) | 639 | 8.52 (7.88-9.22) |
| Alanine aminotransferase increased | Investigations | 26 | 5.94 (4.03-8.75) | 74 | 3.91 (3.11-4.92) | 192 | 2.69 (2.33-3.10) | 204 | 3.05 (2.66-3.51) | 496 | 2.88 (2.63-3.15) |
| Aspartate aminotransferase increased | Investigations | 17 | 4.28 (2.66-6.91) | 63 | 4.13 (3.22-5.29) | 183 | 2.98 (2.57-3.45) | 203 | 3.21 (2.79-3.68) | 466 | 3.09 (2.82-3.39) |
| Hair colour changes | Skin and subcutaneous tissue disorders | 11 | 77.48 (41.81-143.58) | 67 | 27.09 (21.20-34.60) | 156 | 7.43 (6.33-8.71) | 78 | 7.00 (5.59-8.78) | 312 | 9.36 (8.36-10.48) |
| Metastases to bone | Neoplasms benign, malignant and unspecified (incl cysts and polyps) | 3 | 43.13 (13.54-137.44) | 14 | 8.66 (5.11-14.68) | 95 | 5.18 (4.23-6.35) | 102 | 4.19 (3.44-5.09) | 214 | 5.61 (4.90-6.43) |
| Metastases to lung | Neoplasms benign, malignant and unspecified (incl cysts and polyps) | 4 | 26.85 (9.92-72.63) | 20 | 12.44 (7.99-19.37) | 72 | 5.28 (4.18-6.67) | 74 | 4.41 (3.50-5.55) | 170 | 5.93 (5.09-6.90) |
| Blood thyroid stimulating hormone increased | Investigations | 9 | 35.22 (18.08-68.62) | 14 | 7.07 (4.18-11.98) | 83 | 7.85 (6.31-9.77) | 63 | 5.76 (4.49-7.41) | 169 | 7.37 (6.32-8.58) |
| Hypercholesterolaemia | Metabolism and nutrition disorders | 9 | 54.48 (27.77-106.86) | 24 | 7.98 (5.33-11.94) | 66 | 6.33 (4.96-8.08) | 47 | 5.68 (4.25-7.59) | 146 | 6.32 (5.36-7.45) |
| Hypertriglyceridaemia | Metabolism and nutrition disorders | 7 | 8.67 (4.12-18.28) | 18 | 8.23 (5.17-13.10) | 44 | 7.17 (5.31-9.67) | 32 | 11.22 (7.86-16.03) | 101 | 6.51 (5.34-7.93) |
| Lipase increased | Investigations | 6 | 13.81 (6.16-30.95) | 13 | 7.92 (4.58-13.69) | 35 | 3.93 (2.81-5.48) | 41 | 4.60 (3.38-6.28) | 95 | 4.80 (3.92-5.88) |

**Abbreviations**: CI, confidence interval; PT, preferred term; ROR, reporting odds ratio; SOC, system organ class.

**Table S7.** Matrix of positive preferred-term signals across the seven study drugs.

The complete table is provided in Supplementary Data Sheet 1, worksheet “Table S7”.

**Table S8.** Formal analysis of age-related reporting heterogeneity.

The complete table is provided in Supplementary Data Sheet 1, worksheet “Table S8”.

**Table S9.** Pairwise positive-PT spectrum and target-set similarity across the seven study drugs.

| **Drug 1** | **Drug 2** | **Positive-PT-spectrum Jaccard** | **Shared positive PTs, n** | **Union positive PTs, n** | **Shared targets** | **Shared targets, n** | **Target union, n** | **Target Jaccard** |
| --- | --- | --- | --- | --- | --- | --- | --- | --- |
| Brigatinib | Ceritinib | 0.288 | 45 | 156 | ALK; ROS1; IGF-1R | 3 | 5 | 0.600 |
| Crizotinib | Ceritinib | 0.214 | 45 | 210 | ALK; ROS1 | 2 | 4 | 0.500 |
| Crizotinib | Brigatinib | 0.136 | 28 | 206 | ALK; ROS1 | 2 | 6 | 0.333 |
| Crizotinib | Lorlatinib | 0.123 | 34 | 276 | ALK; ROS1 | 2 | 3 | 0.667 |
| Brigatinib | Lorlatinib | 0.103 | 24 | 232 | ALK; ROS1 | 2 | 5 | 0.400 |
| Brigatinib | Cabozantinib | 0.101 | 30 | 297 | FLT3 | 1 | 10 | 0.100 |
| Entrectinib | Brigatinib | 0.100 | 14 | 140 | ROS1; ALK | 2 | 6 | 0.333 |
| Lorlatinib | Ceritinib | 0.099 | 25 | 252 | ALK; ROS1 | 2 | 3 | 0.667 |
| Cabozantinib | Ceritinib | 0.091 | 29 | 319 | None | 0 | 9 | 0.000 |
| Crizotinib | Entrectinib | 0.083 | 16 | 192 | ALK; ROS1 | 2 | 4 | 0.500 |
| Entrectinib | Repotrectinib | 0.080 | 6 | 75 | TRKA/B/C; ROS1; ALK | 3 | 3 | 1.000 |
| Entrectinib | Ceritinib | 0.067 | 11 | 164 | ROS1; ALK | 2 | 4 | 0.500 |
| Crizotinib | Cabozantinib | 0.061 | 22 | 359 | MET | 1 | 8 | 0.125 |
| Entrectinib | Lorlatinib | 0.055 | 12 | 218 | ROS1; ALK | 2 | 3 | 0.667 |
| Lorlatinib | Cabozantinib | 0.039 | 15 | 388 | None | 0 | 8 | 0.000 |
| Lorlatinib | Repotrectinib | 0.034 | 6 | 177 | ALK; ROS1 | 2 | 3 | 0.667 |
| Entrectinib | Cabozantinib | 0.031 | 9 | 292 | None | 0 | 9 | 0.000 |
| Brigatinib | Repotrectinib | 0.029 | 3 | 104 | ALK; ROS1 | 2 | 6 | 0.333 |
| Repotrectinib | Ceritinib | 0.024 | 3 | 125 | ROS1; ALK | 2 | 4 | 0.500 |
| Crizotinib | Repotrectinib | 0.019 | 3 | 158 | ALK; ROS1 | 2 | 4 | 0.500 |
| Repotrectinib | Cabozantinib | 0.016 | 4 | 250 | None | 0 | 9 | 0.000 |

**Note: Across 21 drug pairs, target-set similarity was weakly and non-significantly correlated with positive-PT-spectrum similarity (Spearman ρ=0.212, P=0.356). This analysis was exploratory because drug pairs were not statistically independent and target sets represented concise pharmacologic summaries.**

**Table S10.** Age-stratified medium/high-priority positive PTs with temporal context.

The complete table is provided in Supplementary Data Sheet 1, worksheet “Table S10”.

**Table S11.** Drug-stratified medium/high-priority positive PTs with temporal context.

The complete table is provided in Supplementary Data Sheet 1, worksheet “Table S11”.

**Table S12.** Time to onset data flow and drug-by-age summaries.

**Table** **S12a**. Time to onset and Weibull summaries across age strata in the pooled seven-drug cohort.

| **Cohort** | **Age group** | **PS reports, n** | **Valid TTO, n (%)** | **Median TTO, days (IQR)** | **Ordinary Weibull β (95% CI)** | **Right-truncation-corrected β (95% CI)** |
| --- | --- | --- | --- | --- | --- | --- |
| ALL | 0–17 | 649 | 293 (45.1%) | 44 (13–116) | 0.642 (0.591–0.698) | 0.637 (0.585–0.694) |
| ALL | 18–44 | 3,687 | 1,383 (37.5%) | 51 (18–164.5) | 0.675 (0.649–0.702) | 0.668 (0.641–0.695) |
| ALL | 45–64 | 15,047 | 6,144 (40.8%) | 52 (18–163) | 0.674 (0.662–0.687) | 0.667 (0.654–0.680) |
| ALL | ≥65 | 19,096 | 7,990 (41.8%) | 39 (14–120) | 0.667 (0.656–0.677) | 0.661 (0.650–0.672) |
| ALL | All ages | 38,479 | 15,810 (41.1%) | 44 (16–140) | 0.668 (0.661–0.676) | 0.662 (0.654–0.670) |

**Note:** Valid TTO is presented as n (% of PS reports), and median TTO as median (IQR). Ordinary and right-truncation-corrected Weibull estimates are shown as β (95% CI). Weibull models were not fitted when fewer than 30 valid TTO records were available. TTO values ≤0 or >3,650 days were excluded before analysis. β<1 indicates an early-failure reporting pattern. Abbreviations: CI, confidence interval; IQR, interquartile range; PS, primary suspect; TTO, time to onset.

**Table S12b**. Time to onset and Weibull summaries by drug and age group.

| Cohort | Age group | PS reports, n | Valid TTO, n (%) | Median TTO, days (IQR) | Ordinary Weibull β (95% CI) | Right-truncation-corrected β (95% CI) |
| --- | --- | --- | --- | --- | --- | --- |
| Crizotinib | 0–17 | 229 | 110 (48.0%) | 22.5 (10–72.75) | 0.643 (0.560–0.738) | 0.643 (0.560–0.738) |
| Crizotinib | 18–44 | 1,101 | 339 (30.8%) | 65 (26–213.5) | 0.689 (0.637–0.747) | 0.685 (0.631–0.743) |
| Crizotinib | 45–64 | 3,429 | 1,159 (33.8%) | 60 (21–223.5) | 0.651 (0.624–0.680) | 0.646 (0.617–0.675) |
| Crizotinib | ≥65 | 3,722 | 1,165 (31.3%) | 37 (13–144) | 0.581 (0.557–0.606) | 0.576 (0.551–0.601) |
| Entrectinib | 0–17 | 42 | 23 (54.8%) | 151 (27.5–341) | Not estimated (n<30) | Not estimated (n<30) |
| Entrectinib | 18–44 | 111 | 48 (43.2%) | 17 (7–78.75) | 0.490 (0.398–0.604) | 0.467 (0.368–0.593) |
| Entrectinib | 45–64 | 360 | 153 (42.5%) | 27 (8–101) | 0.588 (0.523–0.661) | 0.576 (0.509–0.653) |
| Entrectinib | ≥65 | 425 | 177 (41.6%) | 14 (6–55) | 0.589 (0.530–0.654) | 0.585 (0.525–0.652) |
| Brigatinib | 0–17 | 18 | 6 (33.3%) | 82.5 (31–208.25) | Not estimated (n<30) | Not estimated (n<30) |
| Brigatinib | 18–44 | 290 | 108 (37.2%) | 56 (15.75–241.5) | 0.655 (0.565–0.761) | 0.631 (0.535–0.744) |
| Brigatinib | 45–64 | 836 | 395 (47.2%) | 60 (19–215) | 0.663 (0.615–0.716) | 0.644 (0.592–0.700) |
| Brigatinib | ≥65 | 940 | 488 (51.9%) | 49 (14–177.25) | 0.630 (0.589–0.674) | 0.608 (0.564–0.656) |
| Lorlatinib | 0–17 | 108 | 37 (34.3%) | 59 (11–100) | 0.813 (0.633–1.044) | 0.810 (0.629–1.042) |
| Lorlatinib | 18–44 | 585 | 164 (28.0%) | 51.5 (21–148.25) | 0.638 (0.571–0.713) | 0.617 (0.545–0.698) |
| Lorlatinib | 45–64 | 1,711 | 514 (30.0%) | 58.5 (18–186.5) | 0.653 (0.613–0.697) | 0.633 (0.589–0.680) |
| Lorlatinib | ≥65 | 1,359 | 366 (26.9%) | 43.5 (12–128.5) | 0.609 (0.565–0.658) | 0.597 (0.549–0.648) |
| Repotrectinib | 0–17 | 4 | 3 (75.0%) | 167 (107.5–195.5) | Not estimated (n<30) | Not estimated (n<30) |
| Repotrectinib | 18–44 | 19 | 4 (21.1%) | 26 (9–51) | Not estimated (n<30) | Not estimated (n<30) |
| Repotrectinib | 45–64 | 52 | 9 (17.3%) | 43 (3–102) | Not estimated (n<30) | Not estimated (n<30) |
| Repotrectinib | ≥65 | 70 | 10 (14.3%) | 7.5 (3.25–23) | Not estimated (n<30) | Not estimated (n<30) |
| Cabozantinib | 0–17 | 211 | 89 (42.2%) | 55 (16–108) | 0.815 (0.697–0.954) | 0.813 (0.694–0.952) |
| Cabozantinib | 18–44 | 1,276 | 536 (42.0%) | 43 (17.75–128.25) | 0.742 (0.697–0.790) | 0.739 (0.693–0.787) |
| Cabozantinib | 45–64 | 7,850 | 3,451 (44.0%) | 46 (17–131) | 0.715 (0.698–0.733) | 0.711 (0.693–0.729) |
| Cabozantinib | ≥65 | 12,083 | 5,479 (45.3%) | 40 (16–114.5) | 0.725 (0.711–0.739) | 0.721 (0.707–0.735) |
| Ceritinib | 0–17 | 37 | 25 (67.6%) | 36 (22–134) | Not estimated (n<30) | Not estimated (n<30) |
| Ceritinib | 18–44 | 305 | 184 (60.3%) | 63 (16–242.75) | 0.666 (0.596–0.745) | 0.662 (0.590–0.743) |
| Ceritinib | 45–64 | 809 | 463 (57.2%) | 79 (25–325) | 0.669 (0.623–0.718) | 0.661 (0.613–0.712) |
| Ceritinib | ≥65 | 497 | 305 (61.4%) | 34 (10–142) | 0.541 (0.497–0.588) | 0.534 (0.489–0.583) |
| Crizotinib | All ages | 8,481 | 2,773 (32.7%) | 48 (16–188) | 0.619 (0.602–0.637) | 0.614 (0.597–0.632) |
| Entrectinib | All ages | 938 | 401 (42.8%) | 20 (7–89) | 0.557 (0.519–0.599) | 0.546 (0.506–0.590) |
| Brigatinib | All ages | 2,084 | 997 (47.8%) | 56 (16–208) | 0.646 (0.616–0.677) | 0.624 (0.592–0.658) |
| Lorlatinib | All ages | 3,763 | 1,081 (28.7%) | 53 (15–157) | 0.635 (0.608–0.664) | 0.618 (0.589–0.649) |
| Repotrectinib | All ages | 145 | 26 (17.9%) | 30.5 (3.25–79.5) | Not estimated (n<30) | Not estimated (n<30) |
| Cabozantinib | All ages | 21,420 | 9,555 (44.6%) | 42 (16–121) | 0.723 (0.712–0.733) | 0.718 (0.708–0.729) |
| Ceritinib | All ages | 1,648 | 977 (59.3%) | 60 (16–265) | 0.612 (0.583–0.642) | 0.604 (0.575–0.636) |

**Note:** Valid TTO is presented as n (% of PS reports), and median TTO as median (IQR). Ordinary and right-truncation-corrected Weibull estimates are shown as β (95% CI). Weibull models were not fitted when fewer than 30 valid TTO records were available. TTO values ≤0 or >3,650 days were excluded before analysis. β<1 indicates an early-failure reporting pattern. Abbreviations: CI, confidence interval; IQR, interquartile range; PS, primary suspect; TTO, time to onset.

**Table S12c**. Time to onset and Weibull summaries for prespecified clinical adverse-event groups.

| **Cohort** | **AE group** | **Valid TTO, n** | **Median TTO, days**  **(IQR)** | **Weibull β**  **(95% CI)** | **Included PT terms** |
| --- | --- | --- | --- | --- | --- |
| ALL | Gastrointestinal toxicity | 3,611 | 28 (11–80) | 0.643 (0.628–0.658) | Diarrhoea; Constipation; Nausea; Vomiting; Stomatitis; Abdominal pain |
| ALL | Hepatic dysfunction | 1,211 | 21 (11–49) | 0.697 (0.671–0.725) | Hepatic function abnormal; Alanine aminotransferase increased; Aspartate aminotransferase increased; Transaminases increased; Hepatic enzyme increased; Liver function test increased; Liver function test abnormal |
| ALL | Skin/mucosal toxicity | 1,435 | 28 (14–62) | 0.751 (0.724–0.779) | Palmar-plantar erythrodysaesthesia syndrome; Blister; Rash; Dermatitis; Skin disorder; Hand dermatitis |
| ALL | Fluid retention/effusion | 774 | 40 (15–124.8) | 0.637 (0.605–0.671) | Oedema; Oedema peripheral; Face oedema; Pleural effusion; Pericardial effusion |
| ALL | Pulmonary toxicity | 931 | 34 (11–110) | 0.627 (0.598–0.657) | Pneumonitis; Interstitial lung disease; Pulmonary toxicity; Respiratory failure; Dyspnoea |
| ALL | Metabolic/lipid disorder | 192 | 31 (12.8–98.5) | 0.662 (0.597–0.733) | Hypertriglyceridaemia; Hypercholesterolaemia; Hyperlipidaemia; Blood triglycerides increased; Blood cholesterol increased |
| ALL | Thyroid/endocrine dysfunction | 483 | 32 (15–69) | 0.787 (0.739–0.837) | Hypothyroidism; Hyperthyroidism; Thyroid disorder; Blood thyroid stimulating hormone increased; Blood thyroid stimulating hormone abnormal; Thyroid function test abnormal; Thyroid hormones decreased; Thyroid hormones increased; Thyroiditis |
| ALL | Neurologic/CNS events | 1,092 | 29 (12–68) | 0.647 (0.620–0.675) | Taste disorder; Dizziness; Memory impairment; Cognitive disorder; Mental disorder; Gait disturbance; Neurotoxicity; Neuropathy peripheral |
| ALL | Blood pressure/vascular | 1,218 | 23 (12–55.8) | 0.683 (0.657–0.711) | Hypertension; Blood pressure increased; Hypertensive crisis; Thrombosis |
| Crizotinib | Gastrointestinal toxicity | 378 | 27 (7–83.8) | 0.531 (0.494–0.571) | Diarrhoea; Constipation; Nausea; Vomiting; Stomatitis; Abdominal pain |
| Crizotinib | Hepatic dysfunction | 184 | 25 (9–57.5) | 0.587 (0.531–0.649) | Hepatic function abnormal; Alanine aminotransferase increased; Aspartate aminotransferase increased; Transaminases increased; Hepatic enzyme increased; Liver function test increased; Liver function test abnormal |
| Crizotinib | Skin/mucosal toxicity | 45 | 20 (10–140) | 0.560 (0.452–0.695) | Palmar-plantar erythrodysaesthesia syndrome; Blister; Rash; Dermatitis; Skin disorder; Hand dermatitis |
| Crizotinib | Fluid retention/effusion | 210 | 37 (12–125) | 0.572 (0.519–0.631) | Oedema; Oedema peripheral; Face oedema; Pleural effusion; Pericardial effusion |
| Crizotinib | Pulmonary toxicity | 237 | 26 (8–77) | 0.566 (0.517–0.620) | Pneumonitis; Interstitial lung disease; Pulmonary toxicity; Respiratory failure; Dyspnoea |
| Crizotinib | Metabolic/lipid disorder | 5 | 17 (5–68) | Not estimated (n<30) | Hypertriglyceridaemia; Hypercholesterolaemia; Hyperlipidaemia; Blood triglycerides increased; Blood cholesterol increased |
| Crizotinib | Thyroid/endocrine dysfunction | 15 | 32 (12–450.5) | Not estimated (n<30) | Hypothyroidism; Hyperthyroidism; Thyroid disorder; Blood thyroid stimulating hormone increased; Blood thyroid stimulating hormone abnormal; Thyroid function test abnormal; Thyroid hormones decreased; Thyroid hormones increased; Thyroiditis |
| Crizotinib | Neurologic/CNS events | 96 | 25 (10–106.8) | 0.538 (0.466–0.620) | Taste disorder; Dizziness; Memory impairment; Cognitive disorder; Mental disorder; Gait disturbance; Neurotoxicity; Neuropathy peripheral |
| Crizotinib | Blood pressure/vascular | 47 | 58 (13.5–260) | 0.554 (0.447–0.687) | Hypertension; Blood pressure increased; Hypertensive crisis; Thrombosis |
| Entrectinib | Gastrointestinal toxicity | 43 | 7 (3–23.5) | 0.559 (0.451–0.694) | Diarrhoea; Constipation; Nausea; Vomiting; Stomatitis; Abdominal pain |
| Entrectinib | Hepatic dysfunction | 18 | 12 (6–36.5) | Not estimated (n<30) | Hepatic function abnormal; Alanine aminotransferase increased; Aspartate aminotransferase increased; Transaminases increased; Hepatic enzyme increased; Liver function test increased; Liver function test abnormal |
| Entrectinib | Skin/mucosal toxicity | 18 | 10 (2.2–21.2) | Not estimated (n<30) | Palmar-plantar erythrodysaesthesia syndrome; Blister; Rash; Dermatitis; Skin disorder; Hand dermatitis |
| Entrectinib | Fluid retention/effusion | 33 | 13 (5–36) | 0.559 (0.437–0.715) | Oedema; Oedema peripheral; Face oedema; Pleural effusion; Pericardial effusion |
| Entrectinib | Pulmonary toxicity | 31 | 13 (6–35) | 0.613 (0.473–0.796) | Pneumonitis; Interstitial lung disease; Pulmonary toxicity; Respiratory failure; Dyspnoea |
| Entrectinib | Metabolic/lipid disorder | 3 | 14 (8.5–108) | Not estimated (n<30) | Hypertriglyceridaemia; Hypercholesterolaemia; Hyperlipidaemia; Blood triglycerides increased; Blood cholesterol increased |
| Entrectinib | Thyroid/endocrine dysfunction | 0 | Not estimable | Not estimated (n<30) | Hypothyroidism; Hyperthyroidism; Thyroid disorder; Blood thyroid stimulating hormone increased; Blood thyroid stimulating hormone abnormal; Thyroid function test abnormal; Thyroid hormones decreased; Thyroid hormones increased; Thyroiditis |
| Entrectinib | Neurologic/CNS events | 79 | 8 (4–28.5) | 0.601 (0.517–0.697) | Taste disorder; Dizziness; Memory impairment; Cognitive disorder; Mental disorder; Gait disturbance; Neurotoxicity; Neuropathy peripheral |
| Entrectinib | Blood pressure/vascular | 2 | 66.5 (34.2–98.8) | Not estimated (n<30) | Hypertension; Blood pressure increased; Hypertensive crisis; Thrombosis |
| Brigatinib | Gastrointestinal toxicity | 171 | 27 (7–123) | 0.565 (0.505–0.632) | Diarrhoea; Constipation; Nausea; Vomiting; Stomatitis; Abdominal pain |
| Brigatinib | Hepatic dysfunction | 79 | 26 (14–73) | 0.706 (0.601–0.828) | Hepatic function abnormal; Alanine aminotransferase increased; Aspartate aminotransferase increased; Transaminases increased; Hepatic enzyme increased; Liver function test increased; Liver function test abnormal |
| Brigatinib | Skin/mucosal toxicity | 41 | 29 (14–139) | 0.565 (0.452–0.706) | Palmar-plantar erythrodysaesthesia syndrome; Blister; Rash; Dermatitis; Skin disorder; Hand dermatitis |
| Brigatinib | Fluid retention/effusion | 56 | 67 (24.8–289) | 0.701 (0.572–0.860) | Oedema; Oedema peripheral; Face oedema; Pleural effusion; Pericardial effusion |
| Brigatinib | Pulmonary toxicity | 113 | 14 (2–62) | 0.509 (0.445–0.584) | Pneumonitis; Interstitial lung disease; Pulmonary toxicity; Respiratory failure; Dyspnoea |
| Brigatinib | Metabolic/lipid disorder | 9 | 233 (61–302) | Not estimated (n<30) | Hypertriglyceridaemia; Hypercholesterolaemia; Hyperlipidaemia; Blood triglycerides increased; Blood cholesterol increased |
| Brigatinib | Thyroid/endocrine dysfunction | 2 | 77 (69–85) | Not estimated (n<30) | Hypothyroidism; Hyperthyroidism; Thyroid disorder; Blood thyroid stimulating hormone increased; Blood thyroid stimulating hormone abnormal; Thyroid function test abnormal; Thyroid hormones decreased; Thyroid hormones increased; Thyroiditis |
| Brigatinib | Neurologic/CNS events | 63 | 47 (15.5–195.5) | 0.616 (0.512–0.741) | Taste disorder; Dizziness; Memory impairment; Cognitive disorder; Mental disorder; Gait disturbance; Neurotoxicity; Neuropathy peripheral |
| Brigatinib | Blood pressure/vascular | 70 | 37 (8.5–168) | 0.610 (0.511–0.729) | Hypertension; Blood pressure increased; Hypertensive crisis; Thrombosis |
| Lorlatinib | Gastrointestinal toxicity | 118 | 31 (11–89.8) | 0.632 (0.556–0.719) | Diarrhoea; Constipation; Nausea; Vomiting; Stomatitis; Abdominal pain |
| Lorlatinib | Hepatic dysfunction | 51 | 25 (10–90) | 0.602 (0.495–0.733) | Hepatic function abnormal; Alanine aminotransferase increased; Aspartate aminotransferase increased; Transaminases increased; Hepatic enzyme increased; Liver function test increased; Liver function test abnormal |
| Lorlatinib | Skin/mucosal toxicity | 21 | 24 (11–78) | Not estimated (n<30) | Palmar-plantar erythrodysaesthesia syndrome; Blister; Rash; Dermatitis; Skin disorder; Hand dermatitis |
| Lorlatinib | Fluid retention/effusion | 176 | 38 (16–109) | 0.687 (0.619–0.764) | Oedema; Oedema peripheral; Face oedema; Pleural effusion; Pericardial effusion |
| Lorlatinib | Pulmonary toxicity | 123 | 54 (18–119.5) | 0.668 (0.587–0.760) | Pneumonitis; Interstitial lung disease; Pulmonary toxicity; Respiratory failure; Dyspnoea |
| Lorlatinib | Metabolic/lipid disorder | 162 | 29.5 (12–97.5) | 0.682 (0.609–0.763) | Hypertriglyceridaemia; Hypercholesterolaemia; Hyperlipidaemia; Blood triglycerides increased; Blood cholesterol increased |
| Lorlatinib | Thyroid/endocrine dysfunction | 28 | 62 (25.5–140.5) | Not estimated (n<30) | Hypothyroidism; Hyperthyroidism; Thyroid disorder; Blood thyroid stimulating hormone increased; Blood thyroid stimulating hormone abnormal; Thyroid function test abnormal; Thyroid hormones decreased; Thyroid hormones increased; Thyroiditis |
| Lorlatinib | Neurologic/CNS events | 137 | 35 (11–96) | 0.613 (0.542–0.692) | Taste disorder; Dizziness; Memory impairment; Cognitive disorder; Mental disorder; Gait disturbance; Neurotoxicity; Neuropathy peripheral |
| Lorlatinib | Blood pressure/vascular | 56 | 65 (21–206.2) | 0.808 (0.659–0.991) | Hypertension; Blood pressure increased; Hypertensive crisis; Thrombosis |
| Repotrectinib | Gastrointestinal toxicity | 4 | 3 (2.8–44) | Not estimated (n<30) | Diarrhoea; Constipation; Nausea; Vomiting; Stomatitis; Abdominal pain |
| Repotrectinib | Hepatic dysfunction | 1 | 10 (10–10) | Not estimated (n<30) | Hepatic function abnormal; Alanine aminotransferase increased; Aspartate aminotransferase increased; Transaminases increased; Hepatic enzyme increased; Liver function test increased; Liver function test abnormal |
| Repotrectinib | Skin/mucosal toxicity | 0 | Not estimable | Not estimated (n<30) | Palmar-plantar erythrodysaesthesia syndrome; Blister; Rash; Dermatitis; Skin disorder; Hand dermatitis |
| Repotrectinib | Fluid retention/effusion | 2 | 13.5 (7.2–19.8) | Not estimated (n<30) | Oedema; Oedema peripheral; Face oedema; Pleural effusion; Pericardial effusion |
| Repotrectinib | Pulmonary toxicity | 0 | Not estimable | Not estimated (n<30) | Pneumonitis; Interstitial lung disease; Pulmonary toxicity; Respiratory failure; Dyspnoea |
| Repotrectinib | Metabolic/lipid disorder | 0 | Not estimable | Not estimated (n<30) | Hypertriglyceridaemia; Hypercholesterolaemia; Hyperlipidaemia; Blood triglycerides increased; Blood cholesterol increased |
| Repotrectinib | Thyroid/endocrine dysfunction | 0 | Not estimable | Not estimated (n<30) | Hypothyroidism; Hyperthyroidism; Thyroid disorder; Blood thyroid stimulating hormone increased; Blood thyroid stimulating hormone abnormal; Thyroid function test abnormal; Thyroid hormones decreased; Thyroid hormones increased; Thyroiditis |
| Repotrectinib | Neurologic/CNS events | 9 | 10 (3–75) | Not estimated (n<30) | Taste disorder; Dizziness; Memory impairment; Cognitive disorder; Mental disorder; Gait disturbance; Neurotoxicity; Neuropathy peripheral |
| Repotrectinib | Blood pressure/vascular | 2 | 85 (44–126) | Not estimated (n<30) | Hypertension; Blood pressure increased; Hypertensive crisis; Thrombosis |
| Cabozantinib | Gastrointestinal toxicity | 2,659 | 30 (13–79) | 0.700 (0.681–0.719) | Diarrhoea; Constipation; Nausea; Vomiting; Stomatitis; Abdominal pain |
| Cabozantinib | Hepatic dysfunction | 784 | 21 (13–44.2) | 0.860 (0.820–0.902) | Hepatic function abnormal; Alanine aminotransferase increased; Aspartate aminotransferase increased; Transaminases increased; Hepatic enzyme increased; Liver function test increased; Liver function test abnormal |
| Cabozantinib | Skin/mucosal toxicity | 1,291 | 28 (14–61) | 0.795 (0.765–0.826) | Palmar-plantar erythrodysaesthesia syndrome; Blister; Rash; Dermatitis; Skin disorder; Hand dermatitis |
| Cabozantinib | Fluid retention/effusion | 263 | 52 (20–169.5) | 0.708 (0.647–0.775) | Oedema; Oedema peripheral; Face oedema; Pleural effusion; Pericardial effusion |
| Cabozantinib | Pulmonary toxicity | 370 | 42 (16–115) | 0.737 (0.684–0.793) | Pneumonitis; Interstitial lung disease; Pulmonary toxicity; Respiratory failure; Dyspnoea |
| Cabozantinib | Metabolic/lipid disorder | 13 | 28 (20–52) | Not estimated (n<30) | Hypertriglyceridaemia; Hypercholesterolaemia; Hyperlipidaemia; Blood triglycerides increased; Blood cholesterol increased |
| Cabozantinib | Thyroid/endocrine dysfunction | 436 | 31 (15–62.2) | 0.831 (0.778–0.887) | Hypothyroidism; Hyperthyroidism; Thyroid disorder; Blood thyroid stimulating hormone increased; Blood thyroid stimulating hormone abnormal; Thyroid function test abnormal; Thyroid hormones decreased; Thyroid hormones increased; Thyroiditis |
| Cabozantinib | Neurologic/CNS events | 674 | 30 (14–57) | 0.745 (0.707–0.786) | Taste disorder; Dizziness; Memory impairment; Cognitive disorder; Mental disorder; Gait disturbance; Neurotoxicity; Neuropathy peripheral |
| Cabozantinib | Blood pressure/vascular | 1,034 | 22 (11–47) | 0.754 (0.723–0.787) | Hypertension; Blood pressure increased; Hypertensive crisis; Thrombosis |
| Ceritinib | Gastrointestinal toxicity | 278 | 15 (4–59.8) | 0.530 (0.487–0.578) | Diarrhoea; Constipation; Nausea; Vomiting; Stomatitis; Abdominal pain |
| Ceritinib | Hepatic dysfunction | 110 | 26 (5.2–92.5) | 0.541 (0.472–0.620) | Hepatic function abnormal; Alanine aminotransferase increased; Aspartate aminotransferase increased; Transaminases increased; Hepatic enzyme increased; Liver function test increased; Liver function test abnormal |
| Ceritinib | Skin/mucosal toxicity | 21 | 15 (4–40) | Not estimated (n<30) | Palmar-plantar erythrodysaesthesia syndrome; Blister; Rash; Dermatitis; Skin disorder; Hand dermatitis |
| Ceritinib | Fluid retention/effusion | 52 | 27 (15–97.8) | 0.614 (0.503–0.751) | Oedema; Oedema peripheral; Face oedema; Pleural effusion; Pericardial effusion |
| Ceritinib | Pulmonary toxicity | 88 | 54 (14–213) | 0.598 (0.510–0.701) | Pneumonitis; Interstitial lung disease; Pulmonary toxicity; Respiratory failure; Dyspnoea |
| Ceritinib | Metabolic/lipid disorder | 0 | Not estimable | Not estimated (n<30) | Hypertriglyceridaemia; Hypercholesterolaemia; Hyperlipidaemia; Blood triglycerides increased; Blood cholesterol increased |
| Ceritinib | Thyroid/endocrine dysfunction | 3 | 20 (13.5–163.5) | Not estimated (n<30) | Hypothyroidism; Hyperthyroidism; Thyroid disorder; Blood thyroid stimulating hormone increased; Blood thyroid stimulating hormone abnormal; Thyroid function test abnormal; Thyroid hormones decreased; Thyroid hormones increased; Thyroiditis |
| Ceritinib | Neurologic/CNS events | 42 | 72 (17–341.5) | 0.623 (0.489–0.794) | Taste disorder; Dizziness; Memory impairment; Cognitive disorder; Mental disorder; Gait disturbance; Neurotoxicity; Neuropathy peripheral |
| Ceritinib | Blood pressure/vascular | 7 | 67 (26–988.5) | Not estimated (n<30) | Hypertension; Blood pressure increased; Hypertensive crisis; Thrombosis |

**Note:** Median TTO is presented as median (IQR), and Weibull β as β (95% CI). β<1 indicates an early-failure reporting pattern. Included PT terms define each prespecified AE group. Abbreviations: AE, adverse event; CI, confidence interval; IQR, interquartile range; PT, preferred term; TTO, time to onset.

**Table S13.** Additional sensitivity and exploratory subgroup analyses.

**Table S13a.** Summary of the sensitivity analysis excluding reports involving prespecified CYP3A-modulating concomitant drugs.

| **Cohort** | **Age group** | **Original reports** | **Retained reports** | **Excluded reports** | **Reference positive PTs** | **Analysis positive PTs** | **Retained positive PTs** | **Lost reference positive PTs** | **New positive PTs** | **Reference signal retention pct** | **Positive set Jaccard** | **Log2 ROR Spearman** | **Excluded report pct** |
| --- | --- | --- | --- | --- | --- | --- | --- | --- | --- | --- | --- | --- | --- |
| Brigatinib | All ages | 2,084 | 2,076 | 8 | 90 | 89 | 88 | 2 | 1 | 97.78 | 0.967 | 1.00 | 0.384 |
| Cabozantinib | All ages | 21,420 | 21,337 | 83 | 237 | 236 | 234 | 3 | 2 | 98.73 | 0.979 | 1.00 | 0.387 |
| Ceritinib | All ages | 1,648 | 1,634 | 14 | 111 | 111 | 111 | 0 | 0 | 100.00 | 1.000 | 1.00 | 0.850 |
| Crizotinib | All ages | 8,481 | 8,445 | 36 | 144 | 145 | 143 | 1 | 2 | 99.31 | 0.979 | 1.00 | 0.424 |
| Entrectinib | All ages | 938 | 935 | 3 | 64 | 64 | 64 | 0 | 0 | 100.00 | 1.000 | 1.00 | 0.320 |
| Lorlatinib | All ages | 3,763 | 3,755 | 8 | 166 | 166 | 164 | 2 | 2 | 98.80 | 0.976 | 1.00 | 0.213 |
| Pooled | 0–17 | 649 | 646 | 3 | 64 | 66 | 62 | 2 | 4 | 96.88 | 0.912 | 1.00 | 0.462 |
| Pooled | 18–44 | 3,687 | 3,670 | 17 | 159 | 160 | 159 | 0 | 1 | 100.00 | 0.994 | 1.00 | 0.461 |
| Pooled | 45–64 | 15,047 | 14,985 | 62 | 230 | 230 | 227 | 3 | 3 | 98.70 | 0.974 | 1.00 | 0.412 |
| Pooled | ≥65 | 19,096 | 19,025 | 71 | 178 | 176 | 175 | 3 | 1 | 98.31 | 0.978 | 1.00 | 0.372 |
| Pooled | All ages | 38,479 | 38,326 | 153 | 299 | 298 | 296 | 3 | 2 | 99.00 | 0.983 | 1.00 | 0.398 |
| Repotrectinib | All ages | 145 | 144 | 1 | 17 | 17 | 17 | 0 | 0 | 100.00 | 1.000 | 1.00 | 0.690 |

**Table S13b.** PT-level changes after exclusion of reports involving prespecified CYP3A-modulating concomitant drugs.

| **Cohort** | **Age group** | **PT name** | **Classification** | **Old a** | **a** | **Old ROR** | **ROR** | **Old RORL** | **RORL** | **Old EBGM05** | **EBGM05** | **Old IC025** | **IC025** |
| --- | --- | --- | --- | --- | --- | --- | --- | --- | --- | --- | --- | --- | --- |
| Pooled | 0-17 | Anaemia | New positive after restriction | 16 | 16 | 3.21 | 3.37 | 1.96 | 2.06 | 1.95 | 2.04 | 0.79 | 0.85 |
| Pooled | 0-17 | Gamma-glutamyltransferase increased | New positive after restriction | 5 | 5 | 4.66 | 5.25 | 1.93 | 2.18 | 1.92 | 2.17 | 0.35 | 0.43 |
| Pooled | 0-17 | Hyperkalaemia | New positive after restriction | 5 | 5 | 4.86 | 5.11 | 2.01 | 2.12 | 2.00 | 2.11 | 0.38 | 0.41 |
| Pooled | 0-17 | Hypokalaemia | New positive after restriction | 8 | 8 | 3.82 | 4.17 | 1.90 | 2.08 | 1.89 | 2.07 | 0.57 | 0.65 |
| Pooled | 0-17 | Optic nerve disorder | Lost positive | 3 | 2 | 30.19 | 19.96 | 9.55 | 4.91 | 9.24 | 4.81 | 0.38 | -0.25 |
| Pooled | 0-17 | Visual impairment | Lost positive | 9 | 8 | 3.99 | 3.61 | 2.07 | 1.80 | 2.06 | 1.79 | 0.70 | 0.51 |
| Pooled | 18-44 | Adrenal insufficiency | New positive after restriction | 8 | 8 | 3.73 | 4.09 | 1.86 | 2.04 | 1.85 | 2.03 | 0.55 | 0.63 |
| Pooled | 45-64 | Cerebral disorder | Lost positive | 20 | 19 | 3.24 | 3.09 | 2.08 | 1.96 | 2.06 | 1.95 | 0.90 | 0.82 |
| Pooled | 45-64 | Hepatic encephalopathy | New positive after restriction | 33 | 33 | 2.75 | 2.86 | 1.95 | 2.03 | 1.94 | 2.02 | 0.88 | 0.93 |
| Pooled | 45-64 | Neuropsychological symptoms | New positive after restriction | 4 | 4 | 5.37 | 5.81 | 1.99 | 2.16 | 1.96 | 2.11 | 0.20 | 0.24 |
| Pooled | 45-64 | Neutrophil percentage increased | New positive after restriction | 7 | 7 | 4.27 | 4.34 | 2.02 | 2.06 | 2.00 | 2.03 | 0.56 | 0.57 |
| Pooled | 45-64 | Skin fissures | Lost positive | 50 | 50 | 2.70 | 2.66 | 2.04 | 2.01 | 2.02 | 2.00 | 0.97 | 0.95 |
| Pooled | 45-64 | Tension | Lost positive | 18 | 17 | 3.42 | 3.24 | 2.15 | 2.01 | 2.12 | 1.99 | 0.92 | 0.83 |
| Pooled | ≥65 | Dermatitis diaper | Lost positive | 3 | 2 | 10.71 | 7.06 | 3.35 | 1.72 | 3.19 | 1.67 | 0.13 | -0.50 |
| Pooled | ≥65 | Faeces soft | Lost positive | 31 | 30 | 2.98 | 2.90 | 2.09 | 2.02 | 2.07 | 2.00 | 0.96 | 0.91 |
| Pooled | ≥65 | Hydrocele | Lost positive | 4 | 4 | 5.55 | 5.51 | 2.05 | 2.04 | 2.01 | 1.99 | 0.21 | 0.20 |
| Pooled | ≥65 | Renal function test abnormal | New positive after restriction | 23 | 23 | 2.97 | 3.08 | 1.97 | 2.04 | 1.95 | 2.02 | 0.85 | 0.89 |
| Cabozantinib | All ages | Faeces pale | New positive after restriction | 12 | 12 | 3.48 | 3.67 | 1.97 | 2.08 | 1.96 | 2.07 | 0.74 | 0.79 |
| Cabozantinib | All ages | Immune-mediated dermatitis | Lost positive | 7 | 7 | 4.35 | 4.25 | 2.07 | 2.02 | 2.05 | 2.00 | 0.58 | 0.56 |
| Cabozantinib | All ages | Penile oedema | Lost positive | 4 | 3 | 7.69 | 5.94 | 2.86 | 1.90 | 2.82 | 1.88 | 0.41 | -0.05 |
| Cabozantinib | All ages | Pulmonary cavitation | New positive after restriction | 6 | 6 | 4.30 | 4.65 | 1.92 | 2.08 | 1.91 | 2.06 | 0.44 | 0.51 |
| Cabozantinib | All ages | Skin hypopigmentation | Lost positive | 10 | 9 | 3.87 | 3.44 | 2.08 | 1.78 | 2.06 | 1.77 | 0.74 | 0.54 |
| Crizotinib | All ages | Gene mutation | New positive after restriction | 4 | 4 | 5.28 | 5.52 | 1.98 | 2.07 | 1.97 | 2.06 | 0.21 | 0.24 |
| Crizotinib | All ages | Liver injury | New positive after restriction | 29 | 29 | 2.84 | 2.92 | 1.97 | 2.03 | 1.97 | 2.03 | 0.89 | 0.93 |
| Crizotinib | All ages | Osteosclerosis | Lost positive | 4 | 3 | 5.80 | 4.40 | 2.17 | 1.42 | 2.16 | 1.41 | 0.27 | -0.20 |
| Pooled | All ages | Blood alkaline phosphatase increased | New positive after restriction | 164 | 161 | 2.33 | 2.36 | 2.00 | 2.02 | 1.98 | 2.01 | 0.97 | 0.99 |
| Pooled | All ages | Blood creatine phosphokinase abnormal | Lost positive | 6 | 5 | 5.16 | 4.41 | 2.30 | 1.82 | 2.26 | 1.80 | 0.58 | 0.29 |
| Pooled | All ages | Blood lactate dehydrogenase abnormal | Lost positive | 6 | 5 | 5.01 | 4.38 | 2.24 | 1.81 | 2.20 | 1.79 | 0.56 | 0.28 |
| Pooled | All ages | Brain oedema | New positive after restriction | 89 | 89 | 2.47 | 2.57 | 2.00 | 2.09 | 1.99 | 2.07 | 0.97 | 1.02 |
| Pooled | All ages | Lung opacity | Lost positive | 22 | 21 | 3.10 | 3.11 | 2.03 | 2.02 | 2.02 | 2.00 | 0.89 | 0.87 |
| Lorlatinib | All ages | Cancer pain | New positive after restriction | 4 | 4 | 5.22 | 5.46 | 1.96 | 2.05 | 1.95 | 2.04 | 0.21 | 0.24 |
| Lorlatinib | All ages | Metastases to bone | New positive after restriction | 14 | 14 | 3.31 | 3.40 | 1.96 | 2.01 | 1.96 | 2.01 | 0.77 | 0.80 |
| Lorlatinib | All ages | Fluid retention | Lost positive | 36 | 36 | 2.79 | 2.77 | 2.01 | 1.99 | 2.01 | 1.99 | 0.94 | 0.92 |
| Lorlatinib | All ages | Hydrocephalus | Lost positive | 7 | 6 | 4.77 | 4.24 | 2.27 | 1.91 | 2.27 | 1.90 | 0.67 | 0.44 |
| Brigatinib | All ages | Blood lactate dehydrogenase abnormal | Lost positive | 3 | 2 | 45.78 | 31.98 | 14.68 | 7.96 | 14.55 | 7.91 | 0.46 | -0.18 |
| Brigatinib | All ages | Blood pressure abnormal | Lost positive | 9 | 9 | 3.87 | 3.84 | 2.01 | 2.00 | 2.01 | 1.99 | 0.67 | 0.66 |
| Brigatinib | All ages | Drug resistance | New positive after restriction | 11 | 11 | 3.50 | 3.98 | 1.93 | 2.20 | 1.93 | 2.20 | 0.70 | 0.84 |

**Table S13c.** Summary of sex-stratified signal analyses.

| **Age group** | **Sex** | **Target reports** | **Reference positive PTs** | **Analysis positive PTs** | **Retained positive PTs** | **Lost reference positive PTs** | **New positive PTs** | **Reference signal retention pct** | **Positive set Jaccard** | **Log2 ROR Spearman** |
| --- | --- | --- | --- | --- | --- | --- | --- | --- | --- | --- |
| 0-17 | F | 280 | 64 | 31 | 28 | 36 | 3 | 43.75 | 0.418 | 0.926 |
| 0-17 | M | 345 | 64 | 55 | 44 | 20 | 11 | 68.75 | 0.587 | 0.952 |
| 18-44 | F | 1,739 | 159 | 117 | 101 | 58 | 16 | 63.52 | 0.577 | 0.943 |
| 18-44 | M | 1,881 | 159 | 106 | 96 | 63 | 10 | 60.38 | 0.568 | 0.926 |
| 45-64 | F | 6,149 | 230 | 175 | 148 | 82 | 27 | 64.35 | 0.576 | 0.918 |
| 45-64 | M | 8,687 | 230 | 155 | 138 | 92 | 17 | 60.00 | 0.559 | 0.922 |
| ≥65 | F | 7,006 | 178 | 136 | 112 | 66 | 24 | 62.92 | 0.554 | 0.902 |
| ≥65 | M | 11,790 | 178 | 147 | 130 | 48 | 17 | 73.03 | 0.667 | 0.935 |
| All ages | F | 15,174 | 299 | 247 | 203 | 96 | 44 | 67.89 | 0.592 | 0.913 |
| All ages | M | 22,703 | 299 | 212 | 197 | 102 | 15 | 65.89 | 0.627 | 0.926 |

**Table S13d.** Concordance of positive PT signals between female and male reports across age strata.

| **Age group** | **Female positive PTs** | **Male positive PTs** | **Shared positive PTs** | **Female only PTs** | **Male only PTs** | **Female male Jaccard** | **Female male log2 ROR Spearman** |
| --- | --- | --- | --- | --- | --- | --- | --- |
| 0-17 | 31 | 55 | 19 | 12 | 36 | 0.284 | 0.817 |
| 18-44 | 117 | 106 | 63 | 54 | 43 | 0.394 | 0.780 |
| 45-64 | 175 | 155 | 84 | 91 | 71 | 0.341 | 0.759 |
| ≥65 | 136 | 147 | 82 | 54 | 65 | 0.408 | 0.772 |
| All ages | 247 | 212 | 144 | 103 | 68 | 0.457 | 0.773 |

**Table S13e.** Summary of reporting-country-stratified signal analyses.

| **Country group** | **Target reports** | **Reference positive PTs** | **Analysis positive PTs** | **Retained positive PTs** | **Lost reference positive PTs** | **New positive PTs** | **Reference signal retention pct** | **Positive set Jaccard** | **Log2 ROR Spearman** |
| --- | --- | --- | --- | --- | --- | --- | --- | --- | --- |
| China | 960 | 299 | 57 | 27 | 272 | 30 | 9.03 | 0.082 | 0.321 |
| Japan | 7,348 | 299 | 87 | 74 | 225 | 13 | 24.75 | 0.237 | 0.632 |
| Other known countries | 12,045 | 299 | 211 | 152 | 147 | 59 | 50.84 | 0.425 | 0.862 |
| United States | 17,837 | 299 | 209 | 163 | 136 | 46 | 54.52 | 0.472 | 0.886 |
| Unknown/missing | 289 | 299 | 24 | 17 | 282 | 7 | 5.69 | 0.056 | 0.419 |

**Table S13f.** Pairwise concordance of positive PT signals across reporting-country strata.

| **Country group 1** | **Country group 2** | **Positive PTs 1** | **Positive PTs 2** | **Shared positive PTs** | **Positive set Jaccard** | **Log2 ROR Spearman** |
| --- | --- | --- | --- | --- | --- | --- |
| United States | Japan | 209 | 87 | 36 | 0.138 | 0.512 |
| United States | China | 209 | 57 | 16 | 0.064 | 0.199 |
| United States | Other known countries | 209 | 211 | 78 | 0.228 | 0.665 |
| Japan | China | 87 | 57 | 15 | 0.116 | 0.419 |
| Japan | Other known countries | 87 | 211 | 45 | 0.178 | 0.559 |
| China | Other known countries | 57 | 211 | 23 | 0.094 | 0.302 |

**Table S13g.** Age-stratified sensitivity analysis after exclusion of cabozantinib reports.

| **Age group** | **Original reports** | **Retained reports** | **Reference positive PTs** | **Analysis positive PTs** | **Retained positive PTs** | **Lost reference positive PTs** | **New positive PTs** | **Reference signal retention pct** | **Positive set Jaccard** | **Log2 ROR Spearman** | **Removed report pct** |
| --- | --- | --- | --- | --- | --- | --- | --- | --- | --- | --- | --- |
| 0-17 | 649 | 438 | 64 | 57 | 45 | 19 | 12 | 70.313 | 0.592 | 0.96 | 32.512 |
| 18-44 | 3,687 | 2,411 | 159 | 132 | 106 | 53 | 26 | 66.667 | 0.573 | 0.95 | 34.608 |
| 45-64 | 15,047 | 7,197 | 230 | 187 | 115 | 115 | 72 | 50.000 | 0.381 | 0.86 | 52.170 |
| ≥65 | 19,096 | 7,013 | 178 | 117 | 57 | 121 | 60 | 32.022 | 0.239 | 0.79 | 63.275 |
| All ages | 38,479 | 17,059 | 299 | 250 | 154 | 145 | 96 | 51.505 | 0.390 | 0.85 | 55.667 |

**Table S13h.** PT-level signal changes after exclusion of cabozantinib reports.

The complete table is provided in Supplementary Data Sheet 1, worksheet “Table S13h”.

**Table S13i.** Distribution of reported indication groups among cabozantinib reports.

| **Indication group** | **Reports** | **Percent** |
| --- | --- | --- |
| Renal cell/renal cancer | 13,921 | 65.0 |
| Hepatocellular/hepatic cancer | 2,250 | 10.5 |
| Other malignancy | 1,942 | 9.1 |
| Non-malignant/unknown indication | 1,775 | 8.3 |
| Thyroid cancer | 1,145 | 5.3 |
| Primary lung cancer/NSCLC | 238 | 1.1 |
| No indication recorded | 149 | 0.7 |

**Table S13j.** Verification of the minimum target-event report criterion across analytical cohorts.

| **Cohort** | **Age group** | **PT rows** | **Positive PTs** | **Positive PTs with a lt 3** | **Minimum a among positive PTs** |
| --- | --- | --- | --- | --- | --- |
| Pooled | All ages | 4486 | 299 | 0 | 3 |
| Pooled | 0-17 | 693 | 64 | 0 | 3 |
| Pooled | 18-44 | 1795 | 159 | 0 | 3 |
| Pooled | 45-64 | 3135 | 230 | 0 | 3 |
| Pooled | ≥65 | 3213 | 178 | 0 | 3 |
| Brigatinib | All ages | 1225 | 90 | 0 | 3 |
| Brigatinib | 0-17 | 60 | 4 | 0 | 3 |
| Brigatinib | 18-44 | 365 | 31 | 0 | 3 |
| Brigatinib | 45-64 | 795 | 48 | 0 | 3 |
| Brigatinib | ≥65 | 795 | 50 | 0 | 3 |
| Entrectinib | All ages | 653 | 64 | 0 | 3 |
| Entrectinib | 0-17 | 84 | 4 | 0 | 3 |
| Entrectinib | 18-44 | 165 | 14 | 0 | 3 |
| Entrectinib | 45-64 | 360 | 34 | 0 | 3 |
| Entrectinib | ≥65 | 401 | 29 | 0 | 3 |
| Cabozantinib | All ages | 3253 | 237 | 0 | 3 |
| Cabozantinib | 0-17 | 307 | 26 | 0 | 3 |
| Cabozantinib | 18-44 | 941 | 90 | 0 | 3 |
| Cabozantinib | 45-64 | 2144 | 179 | 0 | 3 |
| Cabozantinib | ≥65 | 2461 | 172 | 0 | 3 |
| Crizotinib | All ages | 2108 | 144 | 0 | 3 |
| Crizotinib | 0-17 | 351 | 36 | 0 | 3 |
| Crizotinib | 18-44 | 751 | 65 | 0 | 3 |
| Crizotinib | 45-64 | 1385 | 104 | 0 | 3 |
| Crizotinib | ≥65 | 1369 | 83 | 0 | 3 |
| Lorlatinib | All ages | 1816 | 166 | 0 | 3 |
| Lorlatinib | 0–17 | 186 | 16 | 0 | 3 |
| Lorlatinib | 18–44 | 780 | 86 | 0 | 3 |
| Lorlatinib | 45–64 | 1254 | 140 | 0 | 3 |
| Lorlatinib | ≥65 | 1003 | 83 | 0 | 3 |
| Repotrectinib | All ages | 206 | 17 | 0 | 3 |
| Repotrectinib | 0-17 | 21 | 0 | 0 |  |
| Repotrectinib | 18-44 | 37 | 1 | 0 | 3 |
| Repotrectinib | 45-64 | 105 | 10 | 0 | 3 |
| Repotrectinib | ≥65 | 109 | 5 | 0 | 3 |
| Ceritinib | All ages | 1101 | 111 | 0 | 3 |
| Ceritinib | 0–17 | 90 | 10 | 0 | 3 |
| Ceritinib | 18–44 | 391 | 44 | 0 | 3 |
| Ceritinib | 45–64 | 754 | 77 | 0 | 3 |
| Ceritinib | ≥65 | 592 | 53 | 0 | 3 |
